# Supplementary material for: Molecular basis of regio- and stereo-specificity in biosynthesis of bacterial heterodimeric diketopiperazines
Source: Nat Commun. 2020 Dec 7;11:6251. doi: 10.1038/s41467-020-20022-5 (PMC7721796; doi:10.1038/s41467-020-20022-5)
Supplement: Supplementary file 1 — Supplementary Information [file 41467_2020_20022_MOESM1_ESM.pdf]

# Supplementary Information

## Molecular Basis of Regio- and Stereo-Specificity in Biosynthesis of Bacterial Heterodimeric Diketopiperazines

Sun *et al.*

# Table of Contents

## 1. Supplementary Figures

**Supplementary Fig. 1.** Spectra of compound NAS-B

**Supplementary Fig. 2.** Key HMBC and ROESY correlations observed in NAS-B, ASP-A and NAS-E.

**Supplementary Fig. 3.** Spectra of compound ASP-A

**Supplementary Fig. 4.** HPLC analysis of enzymatic conversion of $cW_L$-$P_L$ by mutants in the N-terminal part of NasF5053 and NascB.

**Supplementary Fig. 5.** The eight fragments in the C-terminal part of NascB

**Supplementary Fig. 6.** HPLC analysis of enzymatic conversion of $cW_L$-$P_L$ by mutants on the C-terminal part of NascB.

**Supplementary Fig. 7.** HPLC analysis of enzymatic conversion of $cW_L$-$P_L$ by mutants of NascB in fragment-7.

**Supplementary Fig. 8.** HPLC analysis of the $Nas_{F5053}$ mutants which can improve the production of NAS-B.

**Supplementary Fig. 9.** Spectra of compound NAS-E

**Supplementary Fig. 10.** Spectra of the synthetic NAS-E (TM-3)

**Supplementary Fig. 11.** Crystal structure of $Nas_{F5053}$.

**Supplementary Fig. 12.** Superposition of $Nas_{F5053}$ without the substrates (cyan) and with the substrates (orange).

**Supplementary Fig. 13.** UV-Vis titration and determination of the binding constants of $cW_L$-$P_L$ to $Nas_{F5053}$ (A-C), $Nas_{F5053}$-Q65I-A86G (D-F) and $Nas_{F5053}$-S284A-V288A (G-I).

**Supplementary Fig. 14.** RMSD variation in molecular dynamics simulations.

**Supplementary Fig. 15.** SDS-PAGE analysis of recombinant proteins.

## 2. Supplementary Tables

**Supplementary Table 1.** Product profiles and yields of the P450 reactions using

$cW_L$-$P_L$ as substrate (NasB, NasbB, NascB, $Nas_{F5053}$ and AspB).

**Supplementary Table 2.** NMR data of compound NAS-B in DMSO-$d_6$.

**Supplementary Table 3.** NMR data of compound ASP-A in DMSO-$d_6$.

**Supplementary Table 4.** NMR data of compound NAS-E in DMSO-$d_6$.

**Supplementary Table 5.** X-ray data collection and structure refinement statistics for $Nas_{F5053}$ and its mutants.

**Supplementary Table 6.** Bacterial strains and plasmids.

**Supplementary Table 7.** Primers used in this study.

## 3. Supplementary Methods

3.1. General materials and methods

3.2. Cloning, expression, and purification of P450s and associated proteins

3.3. P450 enzyme assays

3.4. Scaled catalytic reaction and purification of compounds NAS-B and ASP-A

3.5. Site-directed mutagenesis of NasF5053 and NascB

3.6. Construction and screening of NasF5053 mutant libraries

3.7. Synthesis of NAS-E

3.8. Cloning, expression, crystallization and crystal structure determination of NasF5053 and its mutants

3.9. Molecular dynamics simulations

3.10. UV-Vis titration and determination of the binding constants of $cW_L$-$P_L$ to $Nas_{F5053}$, $Nas_{F5053}$-Q65I-A86G or $Nas_{F5053}$-S284A-V288A.

## 4. Supplementary References

# 1. Supplementary Figures

**a**

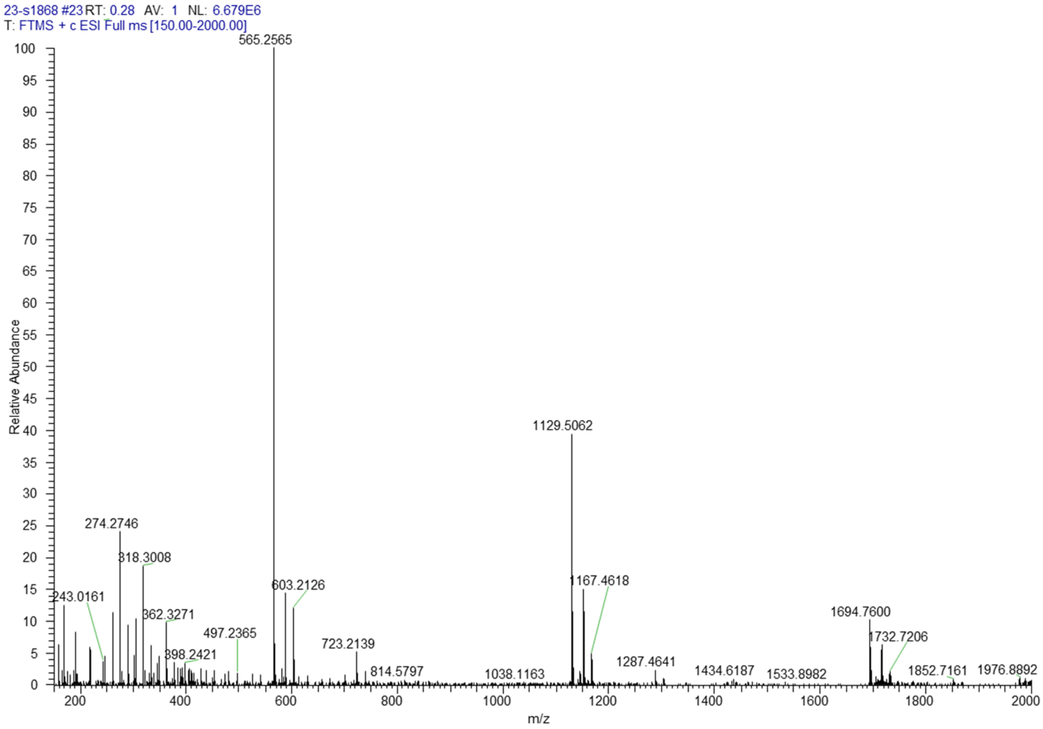


**b**

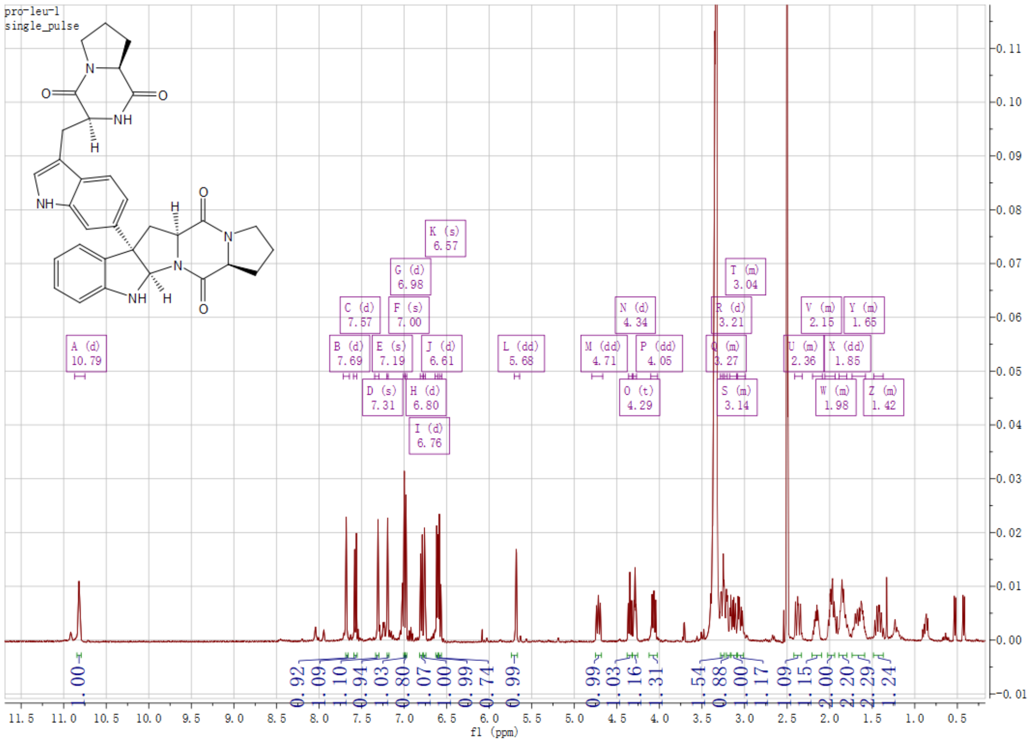

**c**

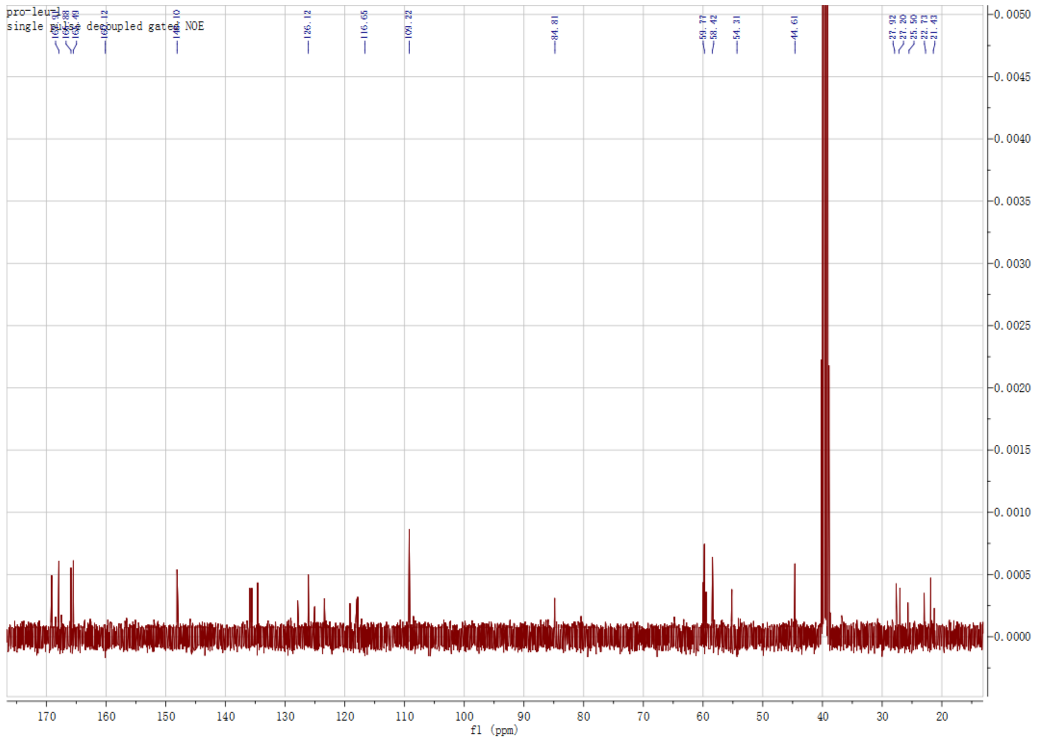
pro-leu-1
single pulse decoupled gated NOE
126.12
116.65
109.22
84.81
59.77
58.42
54.31
44.61
f1 (ppm)

**d**

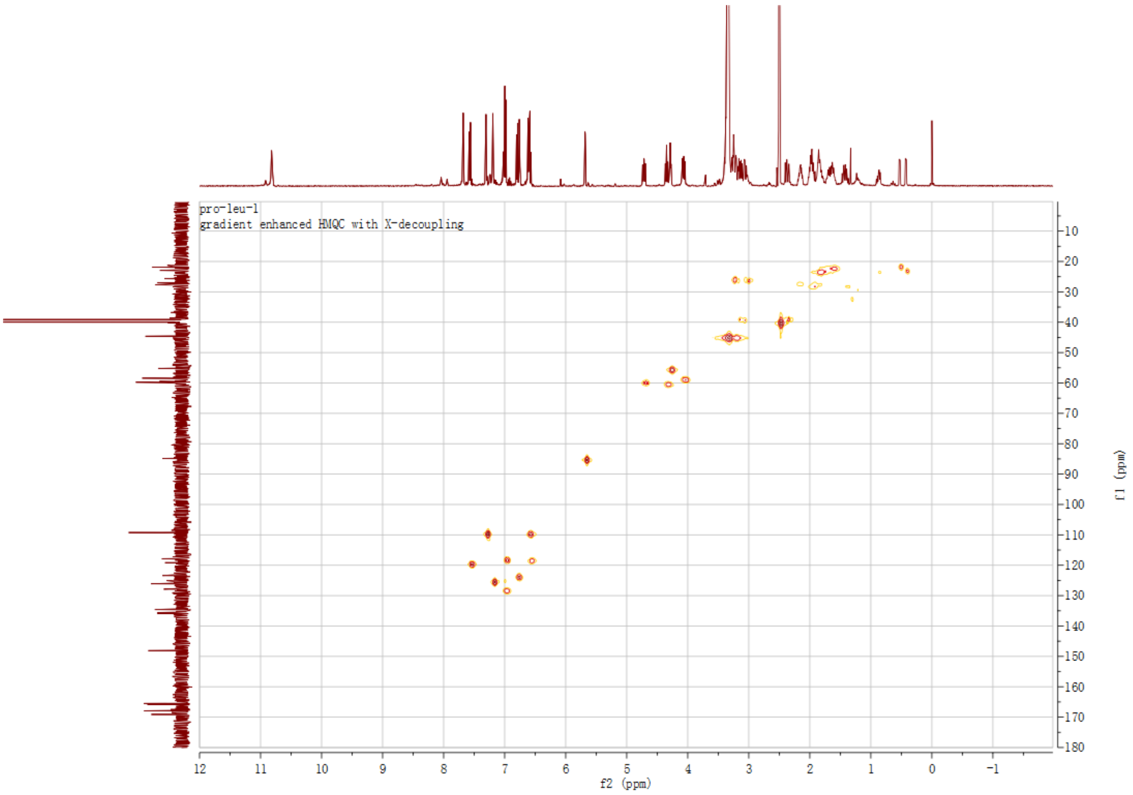
pro-leu-1
gradient enhanced HMQC with X-decoupling
f2 (ppm)
f1 (ppm)

**e**

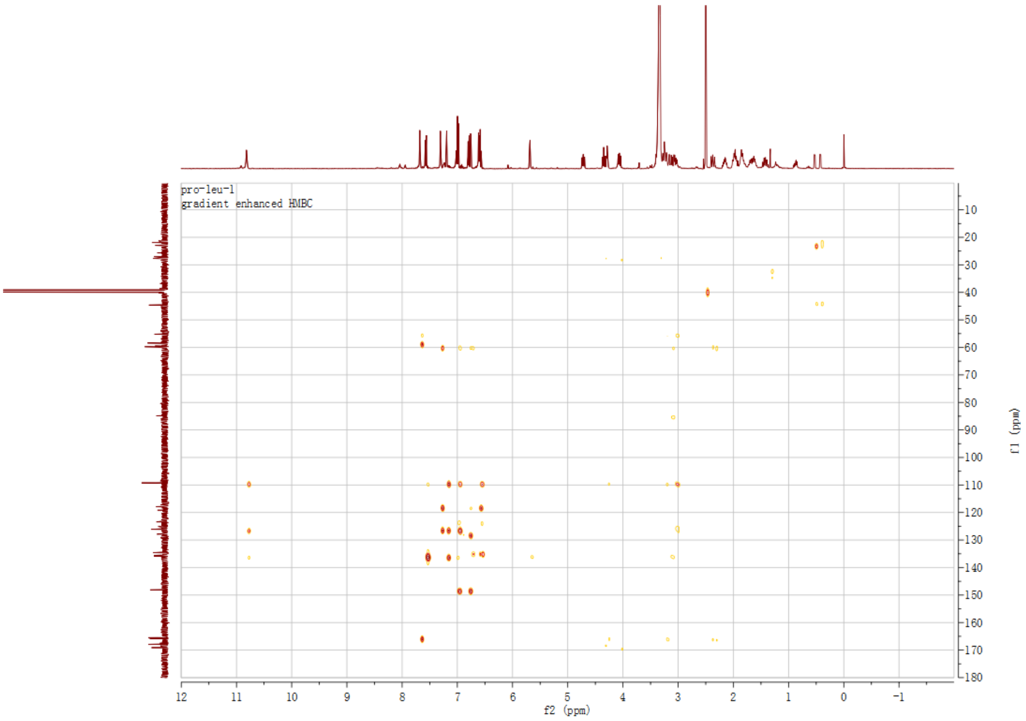


**f**

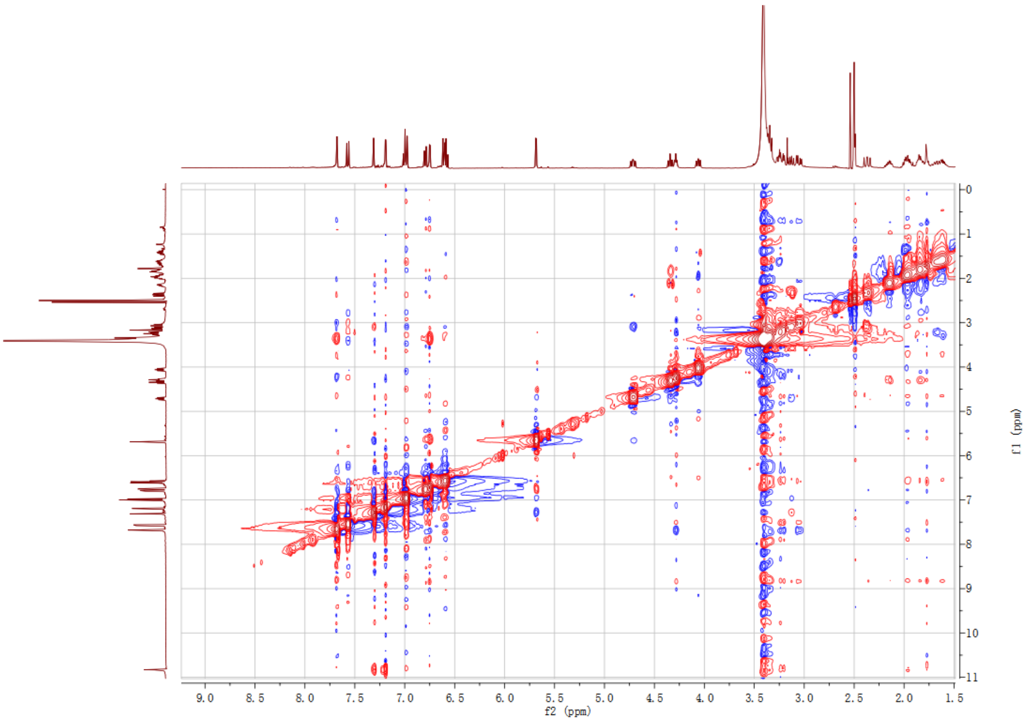


**Supplementary Fig. 1**. Spectra of compound NAS-B. (a) HRESIMS of compound of NAS-B (b) $^{1}$H NMR of compound of NAS-B (c) $^{13}$C NMR of compound of NAS-B (d) HSQC of compound of NAS-B (e) HMBC of compound of NAS-B (f) ROSEY of compound of NAS-B.

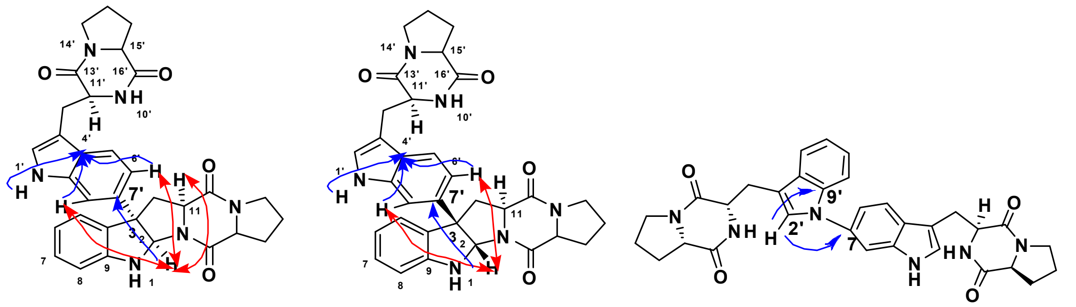

NAS-B NAS-E ASP-A

**Supplementary Fig. 2**. Key HMBC and ROESY correlations observed in NAS-B, ASP-A and NAS-E. (HMBC: blue; ROESY: red).

**a**

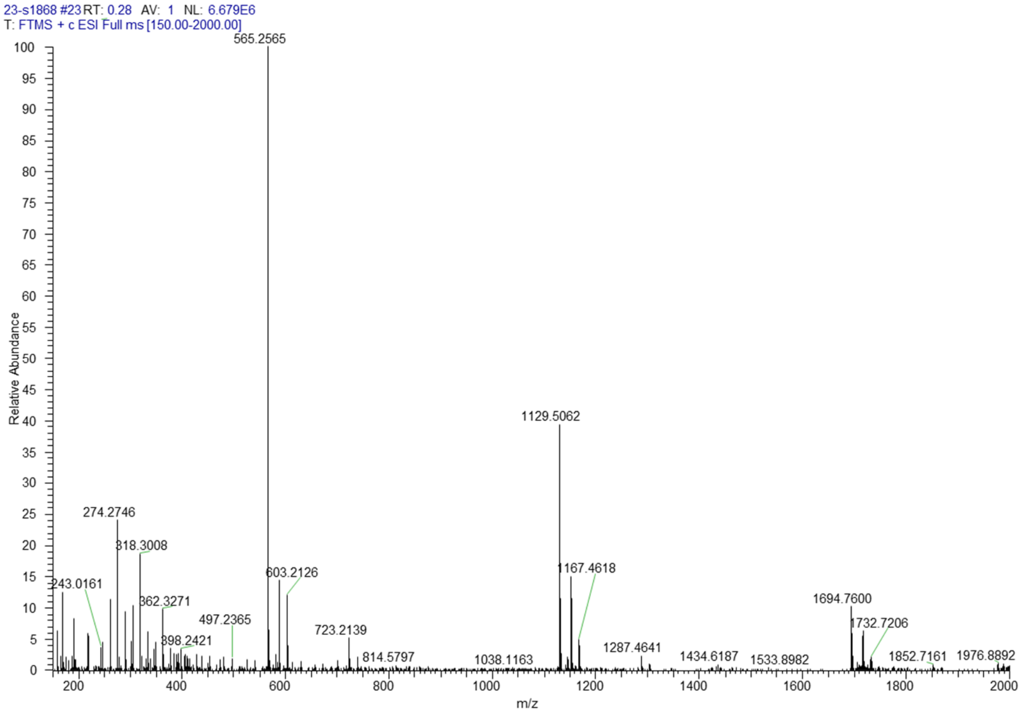
23-s1868 #23 RT: 0.28 AV: 1 NL: 6.679E6
T: FTMS + c ESI Full ms [150.00-2000.00]
565.2565
1129.5062
274.2746
318.3008
243.0161
362.3271
398.2421
497.2365
603.2126
723.2139
814.5797
1038.1163
1167.4618
1287.4641
1434.6187
1533.8982
1694.7600
1732.7206
1852.7161
1976.8892
Relative Abundance
m/z

**b**

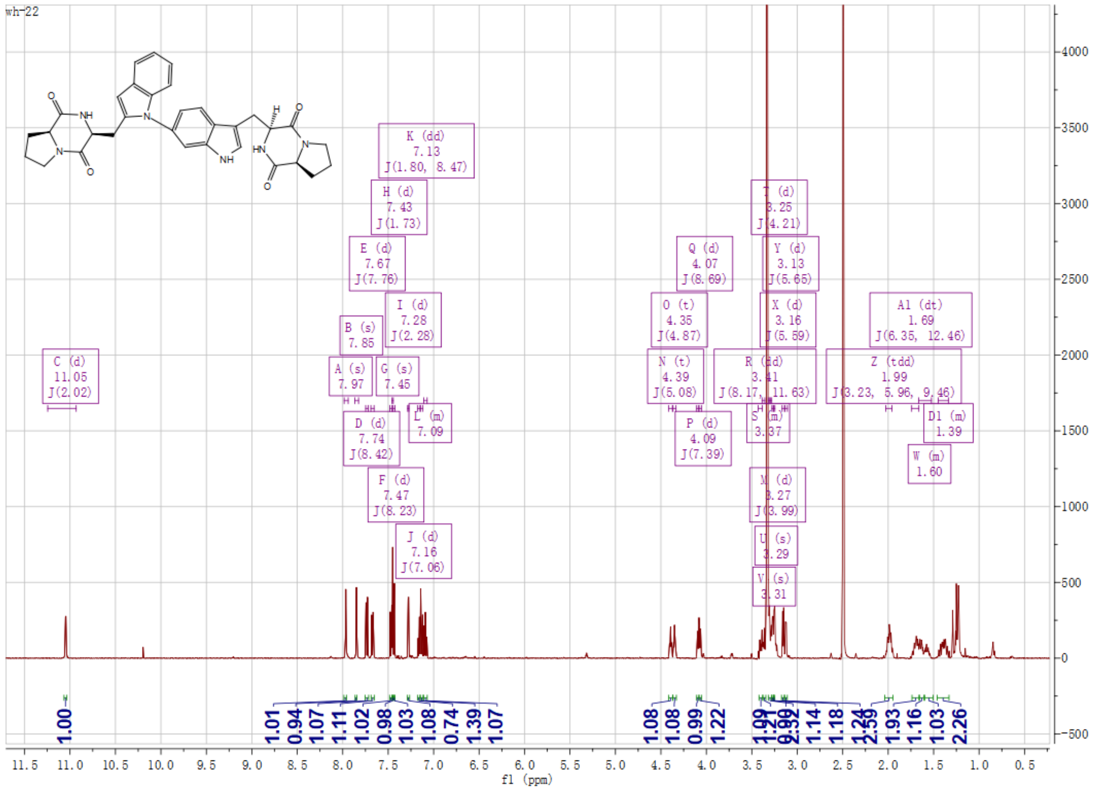
wh-22
K (dd) 7.13 J(1.80, 8.47)
H (d) 7.43 J(1.73)
E (d) 7.67 J(7.76)
I (d) 7.28 J(2.28)
B (s) 7.85
A (s) 7.97
G (s) 7.45
C (d) 11.05 J(2.02)
D (d) 7.74 J(8.42)
L (m) 7.09
F (d) 7.47 J(8.23)
J (d) 7.16 J(7.06)
Q (d) 4.07 J(8.69)
O (t) 4.35 J(4.87)
N (t) 4.39 J(5.08)
P (d) 4.09 J(7.39)
T (d) 3.25 J(4.21)
Y (d) 3.13 J(5.66)
X (d) 3.16 J(5.59)
R (dd) 3.41 J(8.17, 11.63)
S (m) 3.37
M (d) 3.27 J(3.99)
U (s) 3.29
V (s) 3.31
A1 (dt) 1.69 J(6.35, 12.46)
Z (tdd) 1.99 J(3.23, 5.96, 9.46)
D1 (m) 1.39
W (m) 1.60
f1 (ppm)

**c**

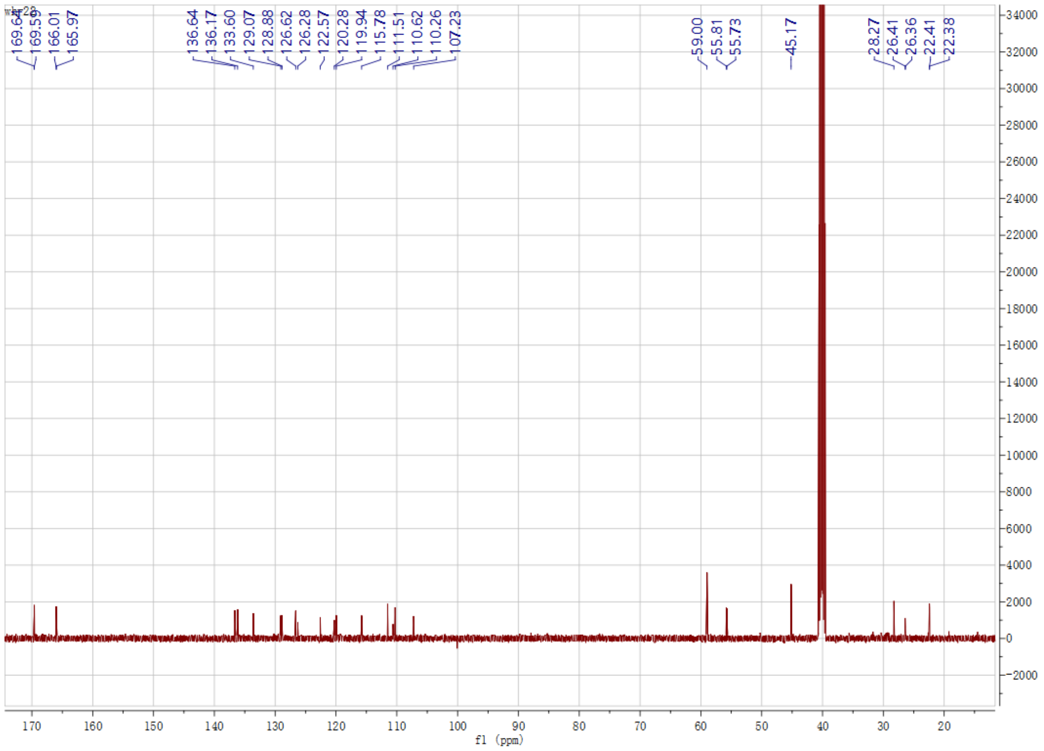
wh-22
169.64
169.59
166.01
165.97
136.64
136.17
133.60
129.07
128.88
126.62
126.28
122.57
120.28
119.94
115.78
111.51
110.62
110.26
107.23
59.00
55.81
55.73
45.17
28.27
26.41
26.36
22.41
22.38
f1 (ppm)

**d**

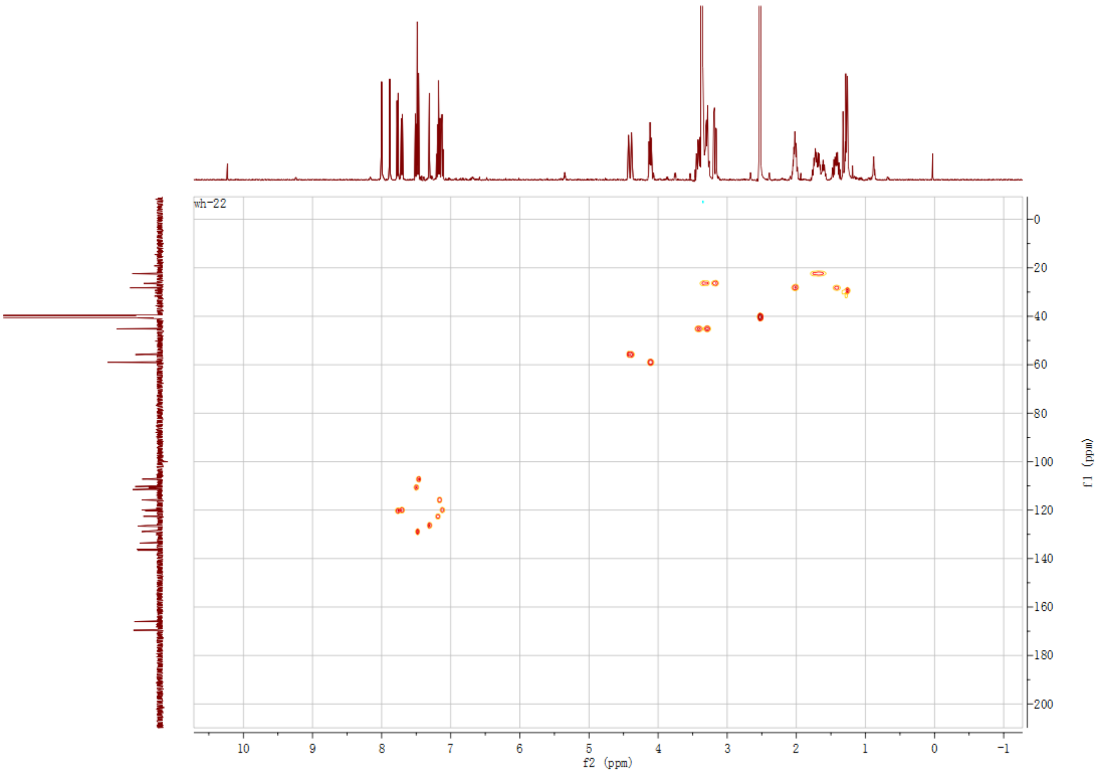
wh-22
f2 (ppm)
f1 (ppm)

**e**

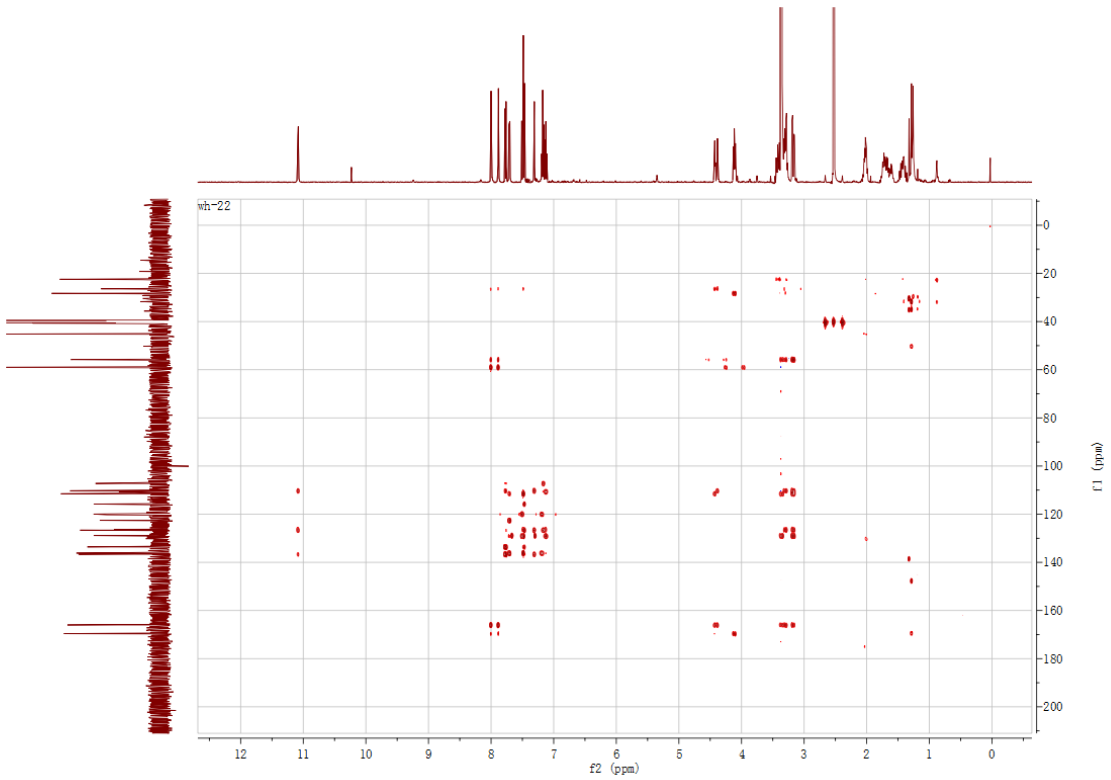


**Supplementary Fig. 3.** Spectra of compound ASP-A. (a) HRESIMS of compound of ASP-A; (b) $^{1}H$ NMR of compound of ASP-A; (c) $^{13}C$ NMR of compound of ASP-A ; (d) HSQC of compound of ASP-A; (e) HMBC of compound of ASP-A.

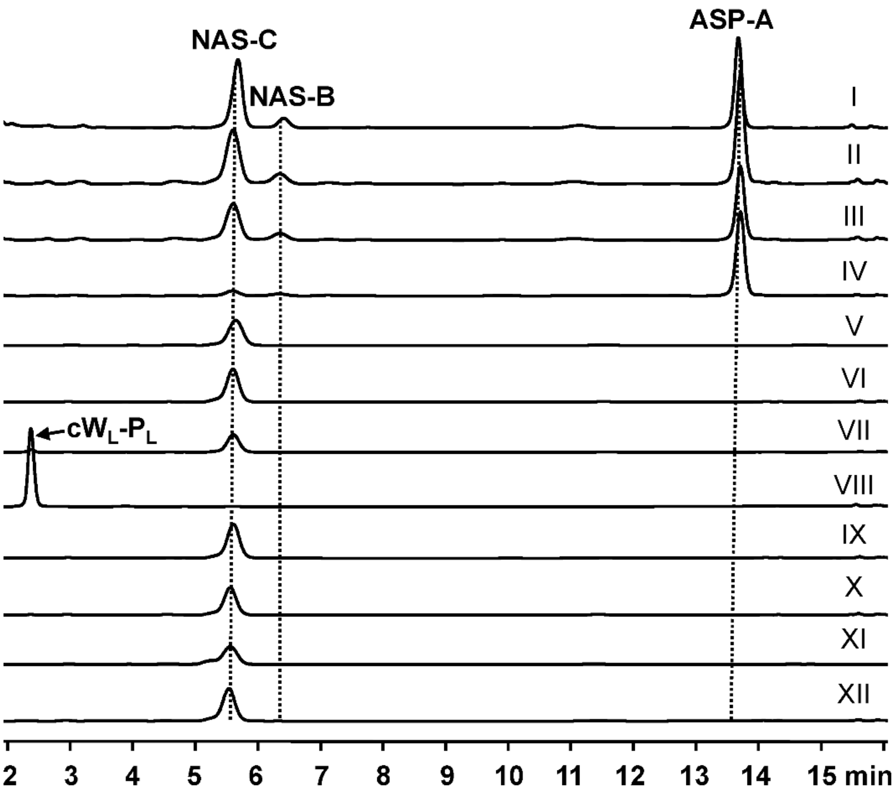


**Supplementary Fig. 4.** HPLC analysis of enzymatic conversion of $cW_L$-$P_L$ by mutants in the N-terminal part of $Nas_{F5053}$ and NascB. I) $Nas_{F5053}$-P43A-T49A-K52E; II) $Nas_{F5053}$-I87V; III) $Nas_{F5053}$-G84A; IV) $Nas_{F5053}$-P43A-T49A-K52E-G84A; V) NascB-P40H; VI) NascB-V44A; VII) NascB-T49A; VIII) NascB-K52E; IX) NascB-S67F; X) NascB-F62L; XI) NascB-G84A; XII) NascB-I87V.

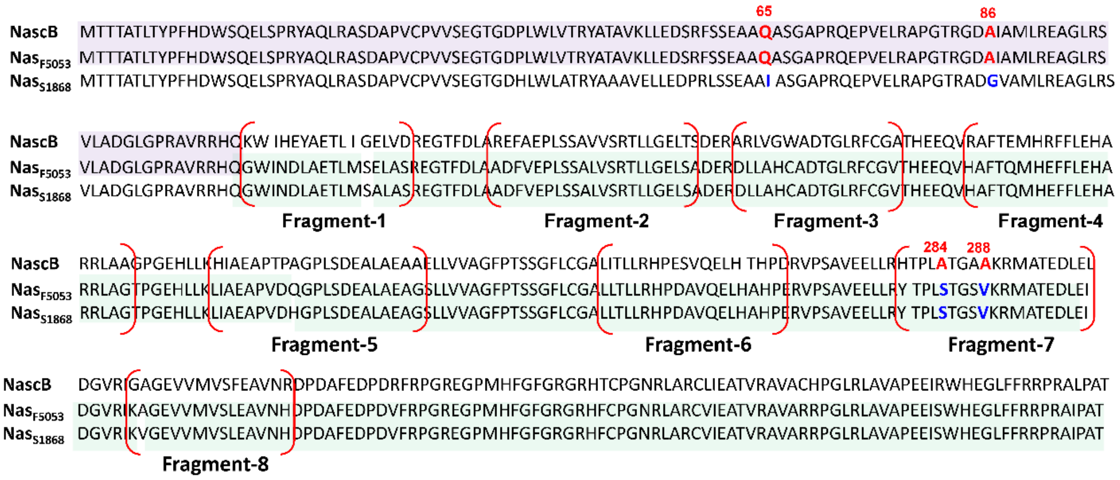


**Supplementary Fig. 5.** The eight fragments in the C-terminal part of NascB

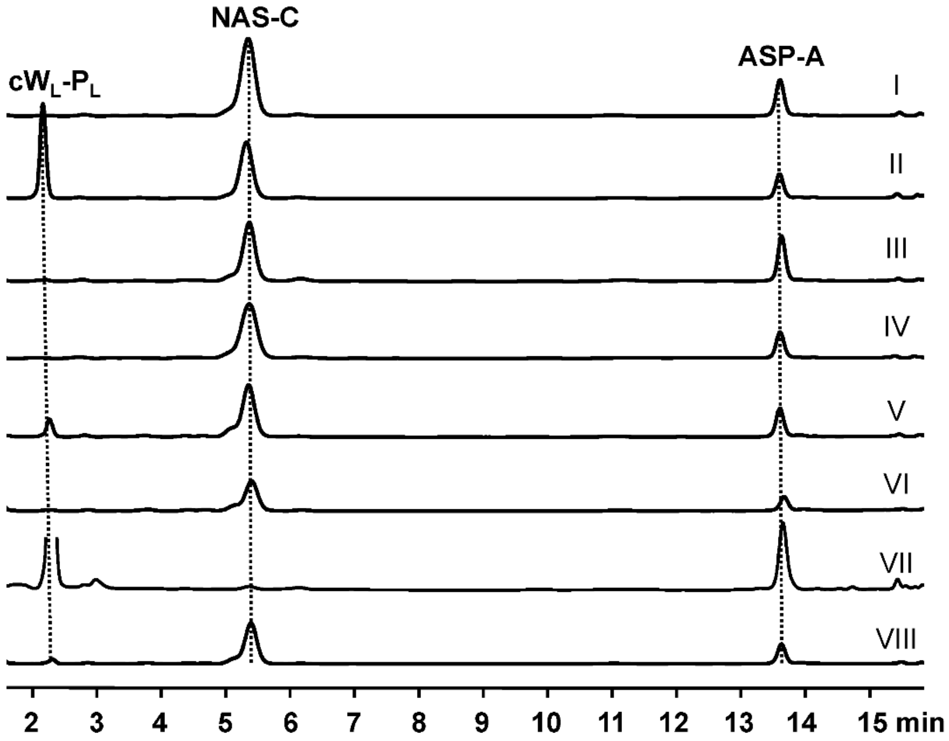


**Supplementary Fig. 6.** HPLC analysis of enzymatic conversion of cW$_L$-P$_L$ by mutants on the C-terminal part of NascB. I) NascB-S1868fragment-1; II) NascB-S1868fragment-2; III) NascB-S1868fragment-3; IV) NascB-S1868fragment-4; V) NascB-S1868fragment-5; VI) NascB-S1868fragment-6; VII) NascB-S1868fragment-7; VIII) NascB-S1868fragment-8.

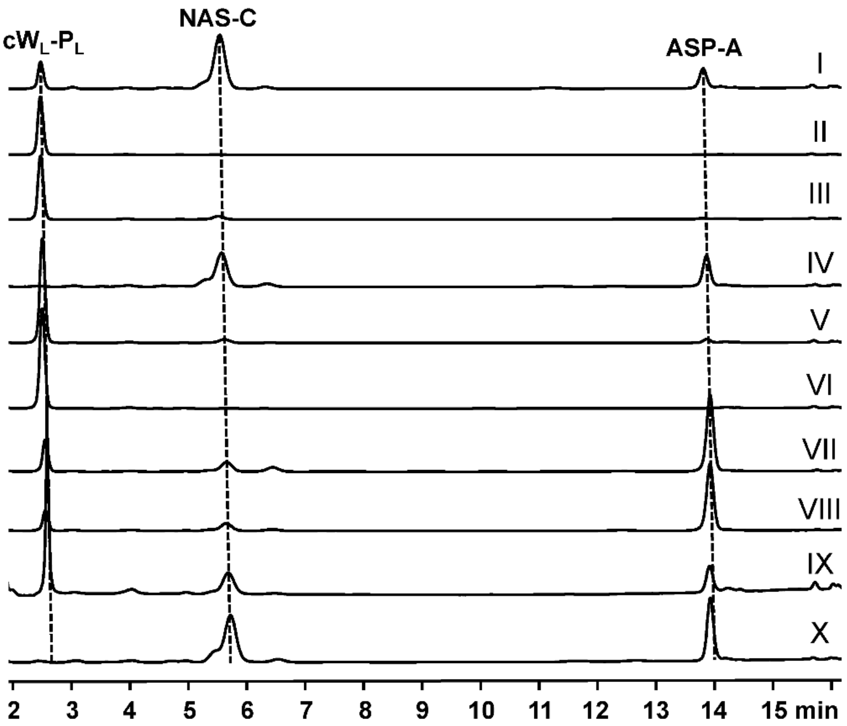


**Supplementary Fig.7.** HPLC analysis of enzymatic conversion of cW$_L$-P$_L$ by mutants of NascB in fragment-7. I) NascB-Q65I-A86G-L298I; II) NascB-Q65I-A86G-H280Y; III) NascB-Q65I-A86G-A288V; IV) NascB-Q65I-A86G-A287S; V) NascB-Q65I-A86G-A284S; VI) NascB-S1868fragment-7-Y280H; VII) NascB-S1868fragment-7-S287A; VIII) NascB-S1868fragment-7-I298L; IX) NascB-S1868fragment-7-S284A; X) NascB-S1868fragment-7-V288A.

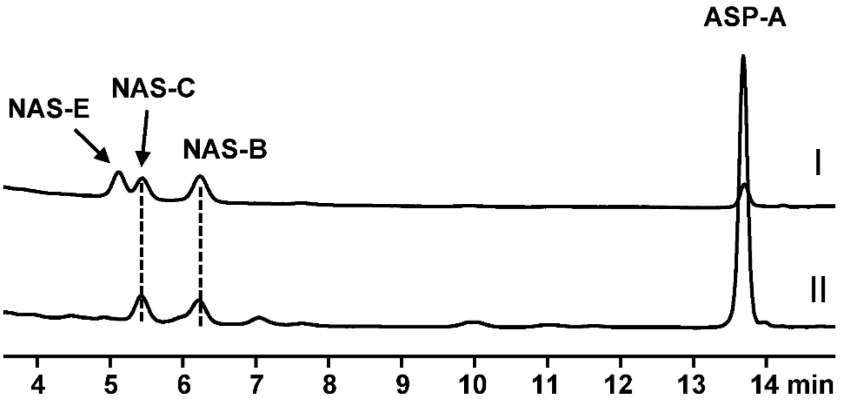


**Supplementary Fig. 8.** HPLC analysis of the Nas$_{F5053}$ mutants which can improve the production of NAS-B. I) Nas$_{F5053}$-A89K; II) Nas$_{F5053}$-V288P.

**a**

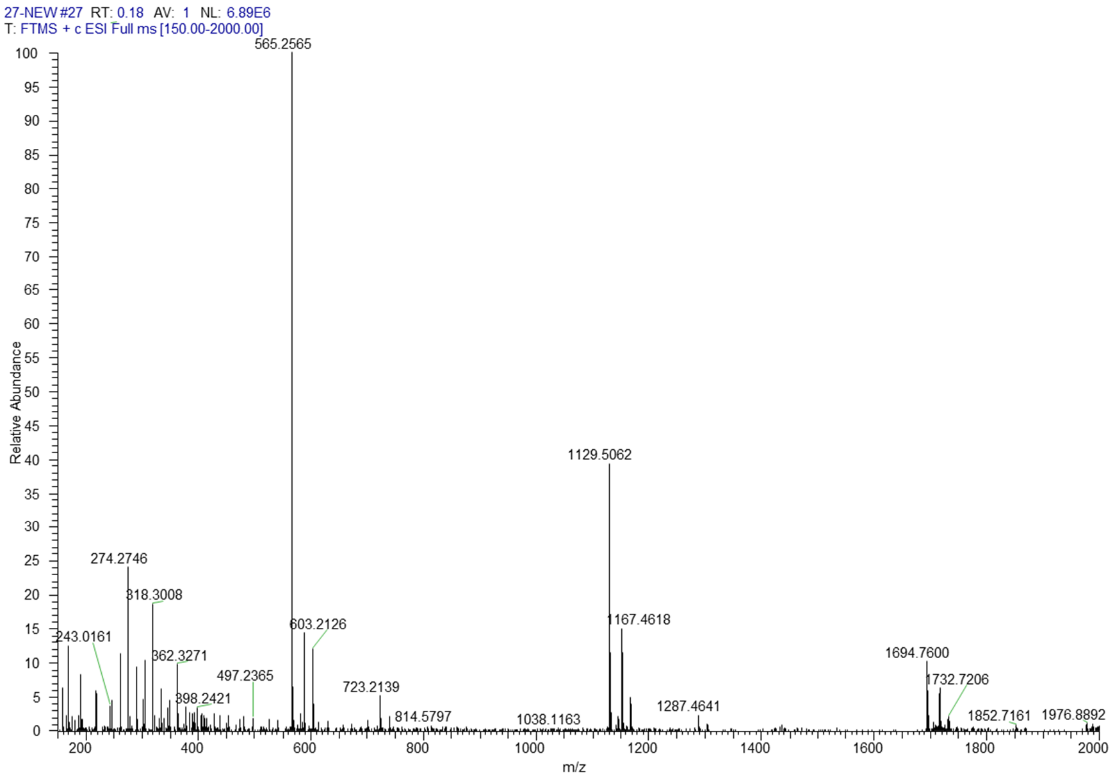

27-NEW #27 RT: 0.18 AV: 1 NL: 6.89E6
T: FTMS + c ESI Full ms [150.00-2000.00]
Relative Abundance
m/z
565.2565
1129.5062
274.2746
318.3008
243.0161
362.3271
398.2421
497.2365
603.2126
723.2139
814.5797
1038.1163
1167.4618
1287.4641
1694.7600
1732.7206
1852.7161
1976.8892


**b**

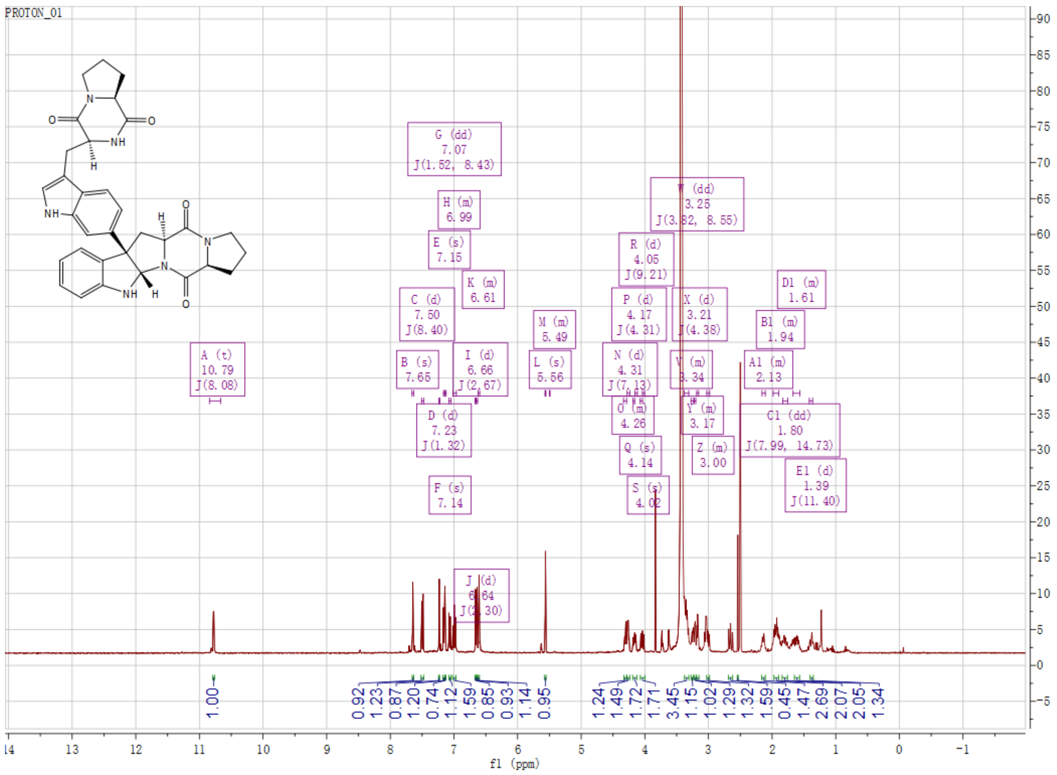

PROTON_01
f1 (ppm)
A (t) 10.79 J(8.08)
B (s) 7.65
C (d) 7.50 J(8.40)
D (d) 7.23 J(1.32)
E (s) 7.15
F (s) 7.14
G (dd) 7.07 J(1.52, 8.43)
H (m) 6.99
I (d) 6.66 J(2.67)
K (m) 6.61
L (s) 5.56
M (m) 5.49
N (d) 4.31 J(7.13)
O (m) 4.26
P (d) 4.17 J(4.31)
Q (s) 4.14
R (d) 4.05 J(9.21)
X (d) 3.21 J(4.38)
Y (m) 3.17
Z (m) 3.00
A1 (m) 2.13
B1 (m) 1.94
C1 (dd) 1.80 J(7.99, 14.73)
D1 (m) 1.61
E1 (d) 1.39 J(11.40)

**c**

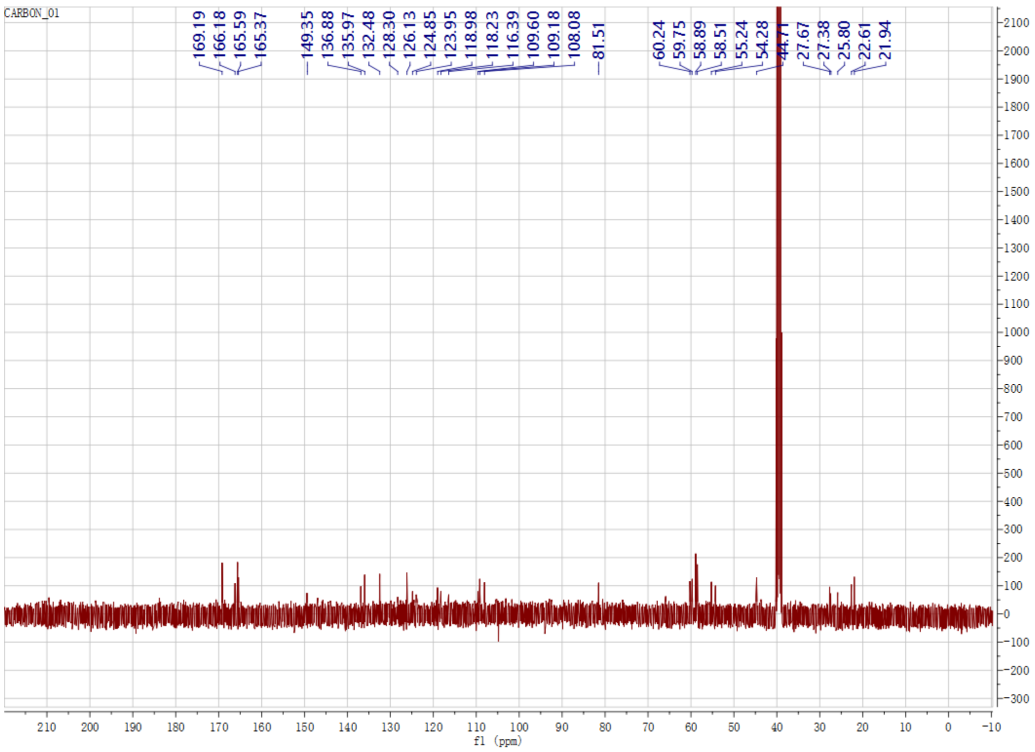

CARBON_01
169.19
166.18
165.59
165.37
149.35
136.88
135.97
132.48
128.30
126.13
124.85
123.95
118.98
118.23
116.39
109.60
109.18
108.08
81.51
60.24
59.75
58.89
58.51
55.24
54.28
44.71
27.67
27.38
25.80
22.61
21.94
f1 (ppm)


**d**

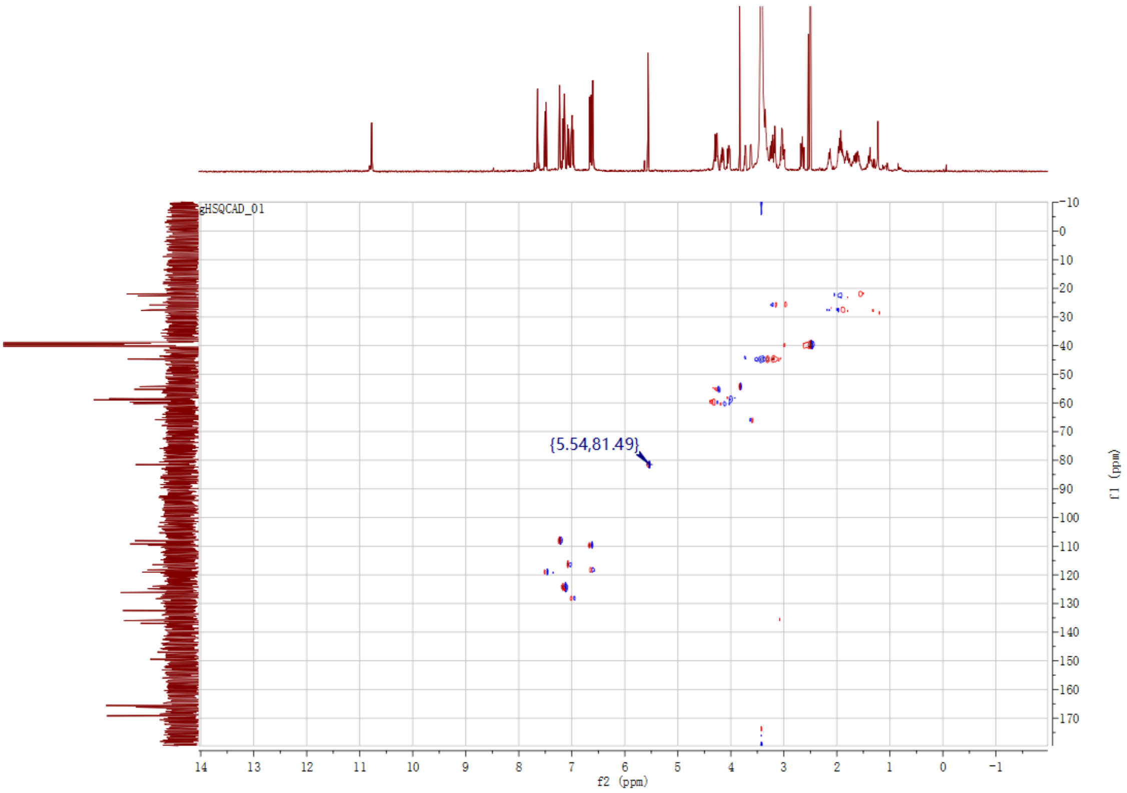

gHSQCAD_01
{5.54,81.49}
f2 (ppm)
f1 (ppm)

**e**

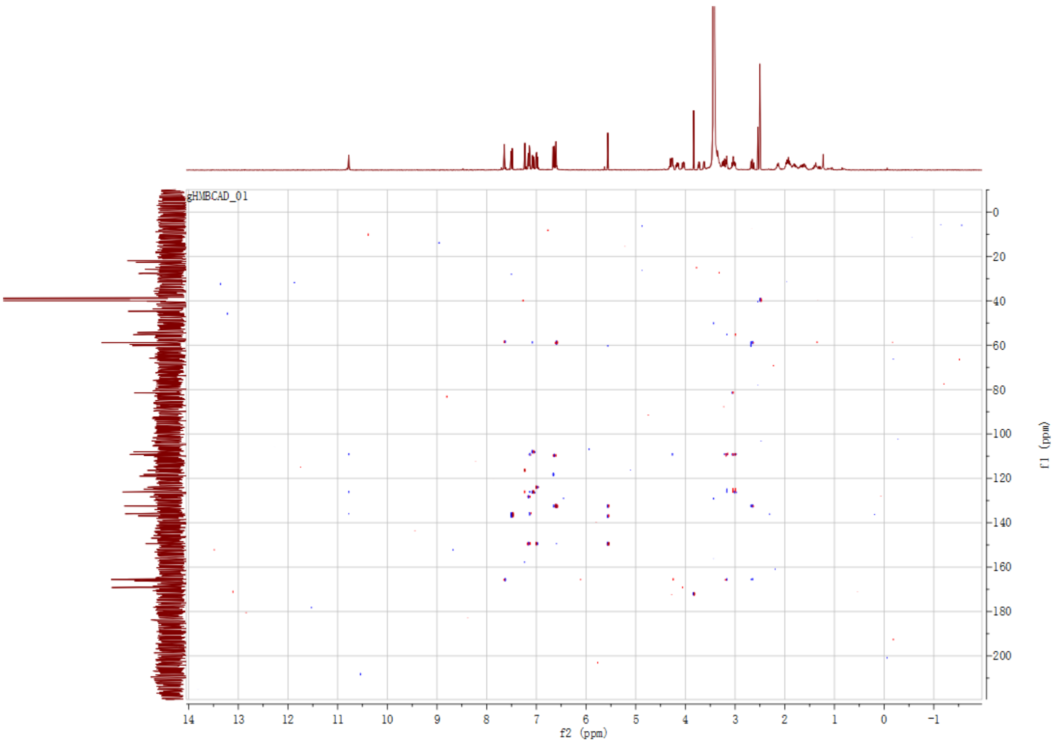


**f**

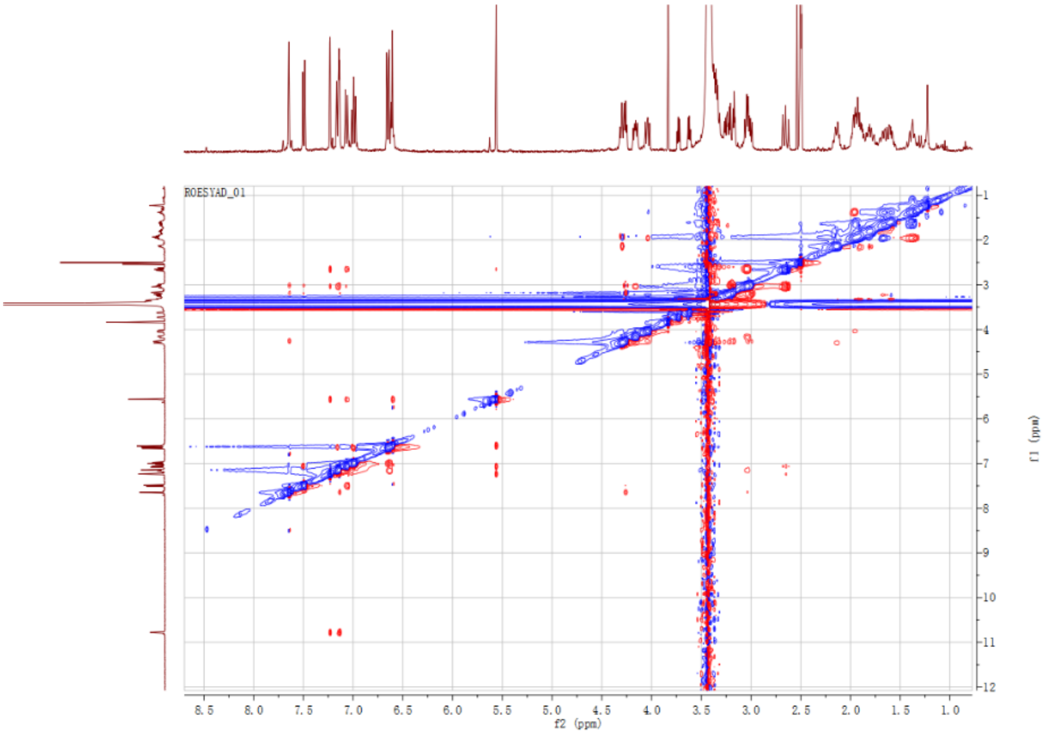


**Supplementary Fig. 9**. Spectra of compound NAS-E. (a) HRESIMS of compound of NAS-E; (b) $^{1}$H NMR of compound of NAS-E; (c) $^{13}$C NMR of compound of NAS-E; (d) HSQC of compound of NAS-E; (e) HMBC of compound of NAS-E; (f) ROSEY of compound of NAS-E.

**a**

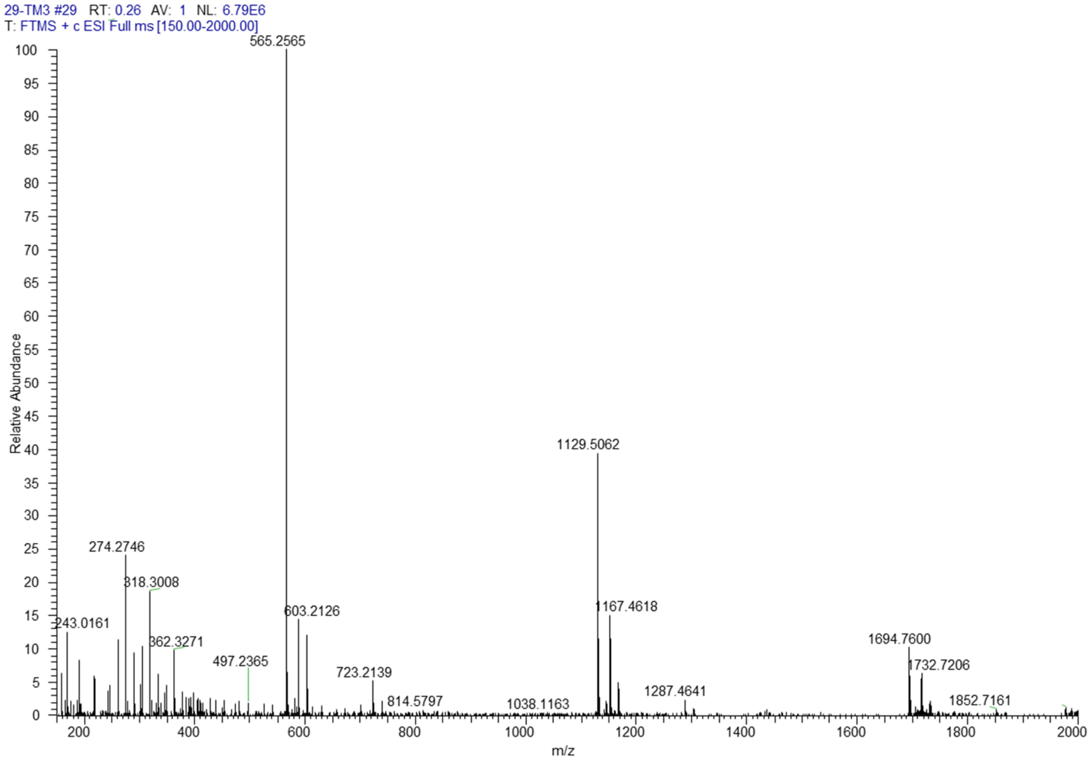

29-TM3 #29 RT: 0.26 AV: 1 NL: 6.79E6
T: FTMS + c ESI Full ms [150.00-2000.00]
565.2565
1129.5062
274.2746
318.3008
243.0161
362.3271
497.2365
603.2126
723.2139
814.5797
1038.1163
1167.4618
1287.4641
1694.7600
1732.7206
1852.7161
Relative Abundance
m/z


**b**

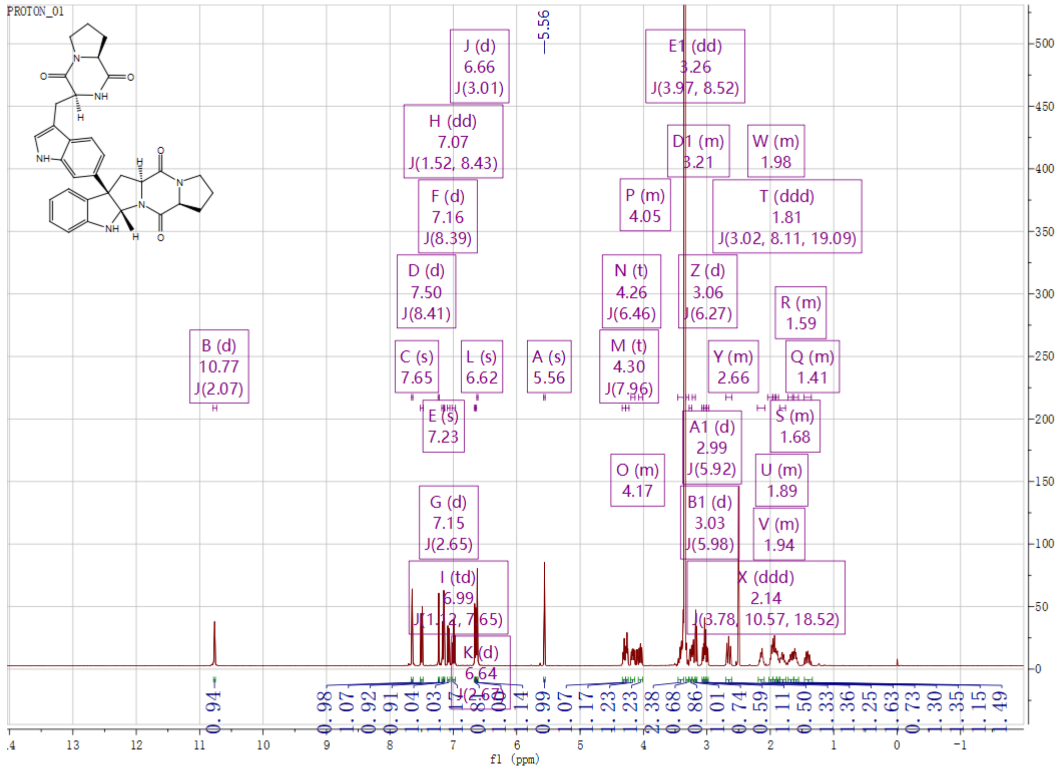

PROTON_01
B (d) 10.77 J(2.07)
C (s) 7.65
D (d) 7.50 J(8.41)
E (s) 7.23
F (d) 7.16 J(8.39)
G (d) 7.15 J(2.65)
H (dd) 7.07 J(1.52, 8.43)
J (d) 6.66 J(3.01)
L (s) 6.62
A (s) 5.56
M (t) 4.30 J(7.96)
N (t) 4.26 J(6.46)
O (m) 4.17
P (m) 4.05
E1 (dd) 3.26 J(3.97, 8.52)
D1 (m) 3.21
Z (d) 3.06 J(6.27)
B1 (d) 3.03 J(5.98)
A1 (d) 2.99 J(5.92)
Y (m) 2.66
X (ddd) 2.14 J(3.78, 10.57, 18.52)
W (m) 1.98
V (m) 1.94
U (m) 1.89
T (ddd) 1.81 J(3.02, 8.11, 19.09)
S (m) 1.68
R (m) 1.59
Q (m) 1.41
f1 (ppm)

**c**

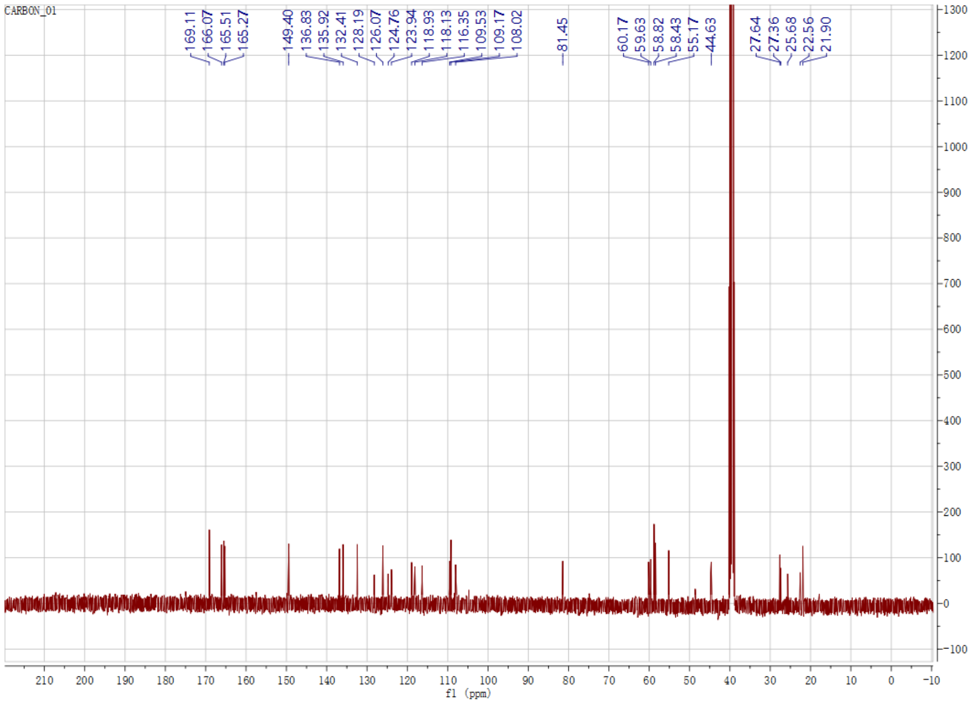
CARBON_01
169.11
166.07
165.51
165.27
149.40
136.83
135.92
132.41
128.19
126.07
124.76
123.94
118.93
118.13
116.35
109.53
109.17
108.02
81.45
60.17
59.63
58.82
58.43
55.17
44.63
27.64
27.36
25.68
22.56
21.90
f1 (ppm)

**d**

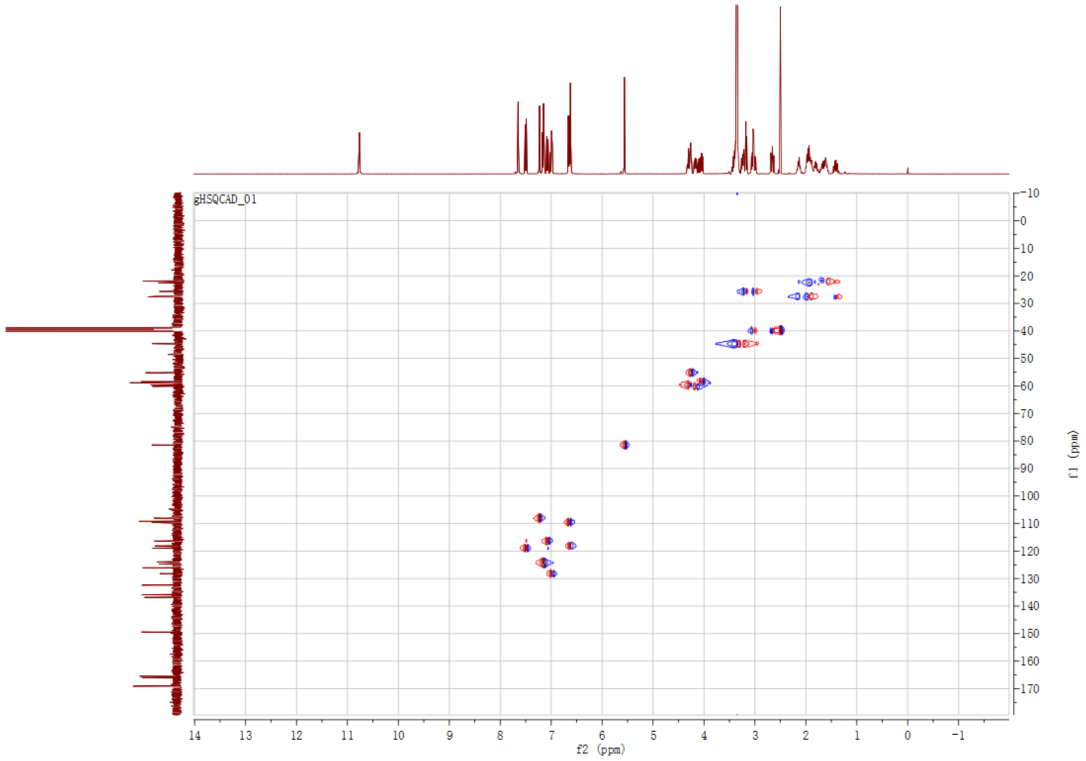
gHSQCAD_01
f2 (ppm)
f1 (ppm)

**e**

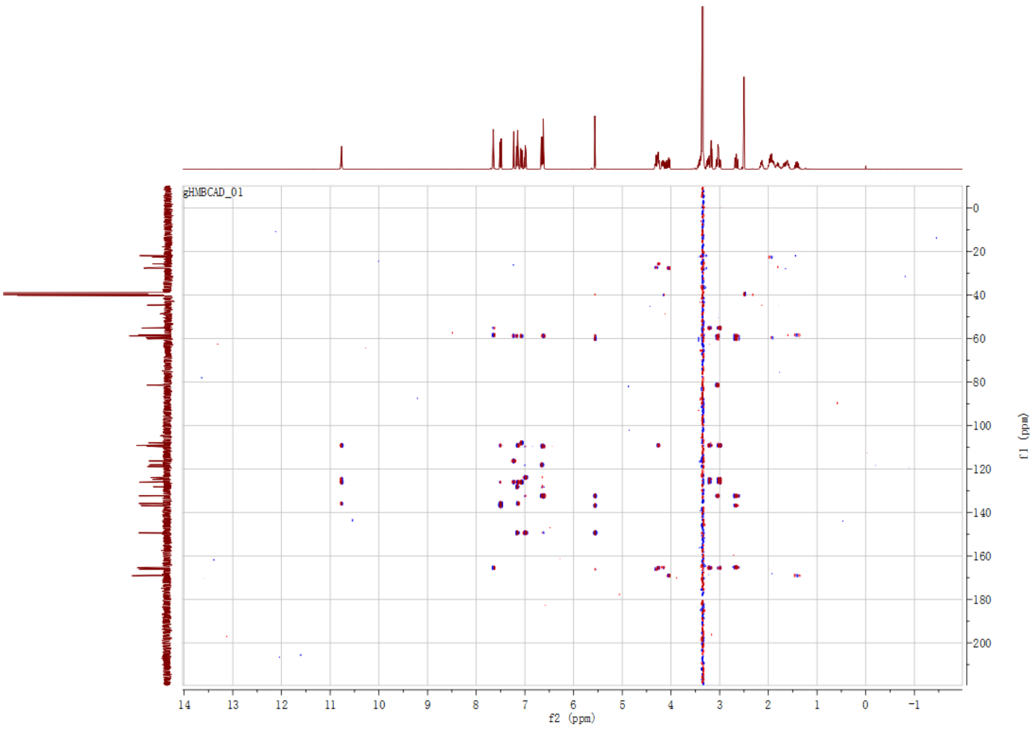


**f**

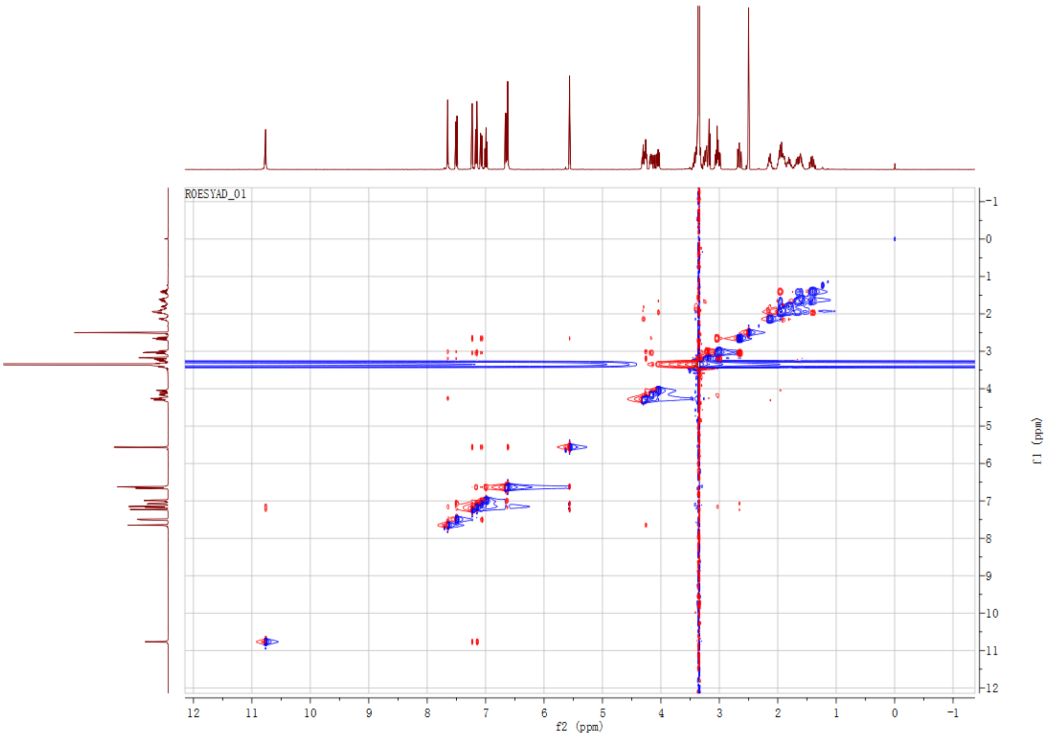


**Supplementary Fig. 10**. Spectra of the synthetic NAS-E (TM-3). (a) HRESIMS of compound of TM-3; (b) $^{1}$H NMR of compound of TM-3; (c) $^{13}$C NMR of compound of TM-3; (d) HSQC of compound of TM-3; (e) HMBC of compound of TM-3; (f) ROSEY of compound of TM-3.

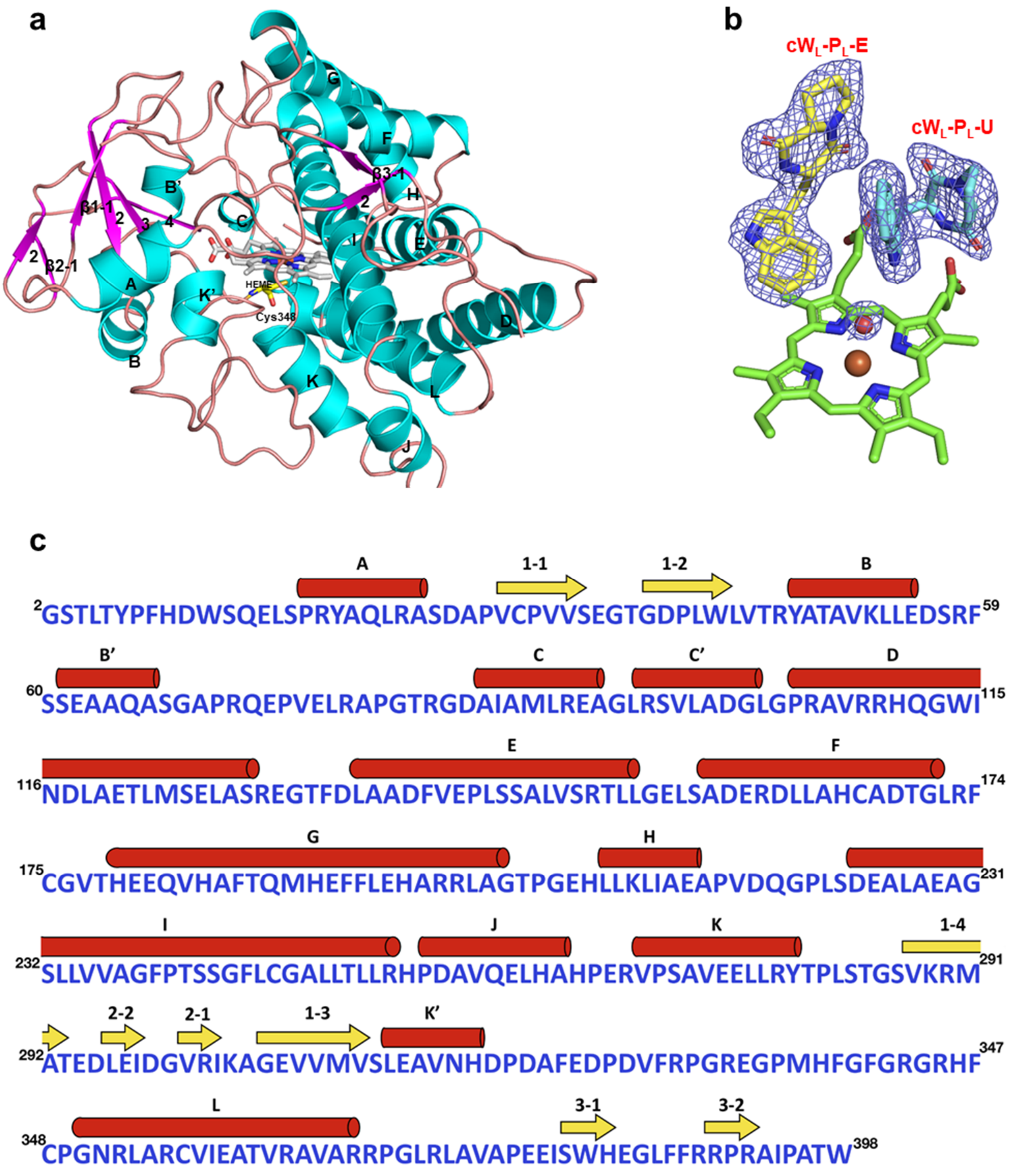


**Supplementary Fig. 11**. Crystal structure of $Nas_{F5053}$. (a) Cartoon representation of the apo structure of $Nas_{F5053}$. The elements of secondary structure and the N- and C-termini are labelled; α-helices are shown in cyan and β-strands in magenta. The iron in the heme is shown as a brown sphere and water molecular is displayed as magenta spheres. Other parts of the heme and $cW_L$-$P_L$-E/ U are displayed as green and yellow sticks, respectively. (b) Fo-Fc electron density omit map in the region of the $cW_L$-$P_L$-E and $cW_L$-$P_L$-U substrates in the $Nas_{F5053}$ complex structure (**Fig. 3a**), contoured at 4σ

level (blue mesh). The map was calculated after 20 cycles of refinement in the absence of the ligands. (c) The numbered amino-acid sequence of Nas$_{F5053}$ used for structure determination. Except for Gly2 and Ser3 added during the cloning process, the rest of the sequence corresponds to the original Nas$_{F5053}$ sequence. Labelled on the top of the sequence is the elements of secondary structure, with yellow arrows for β-strands and red cylinders for α-helices.

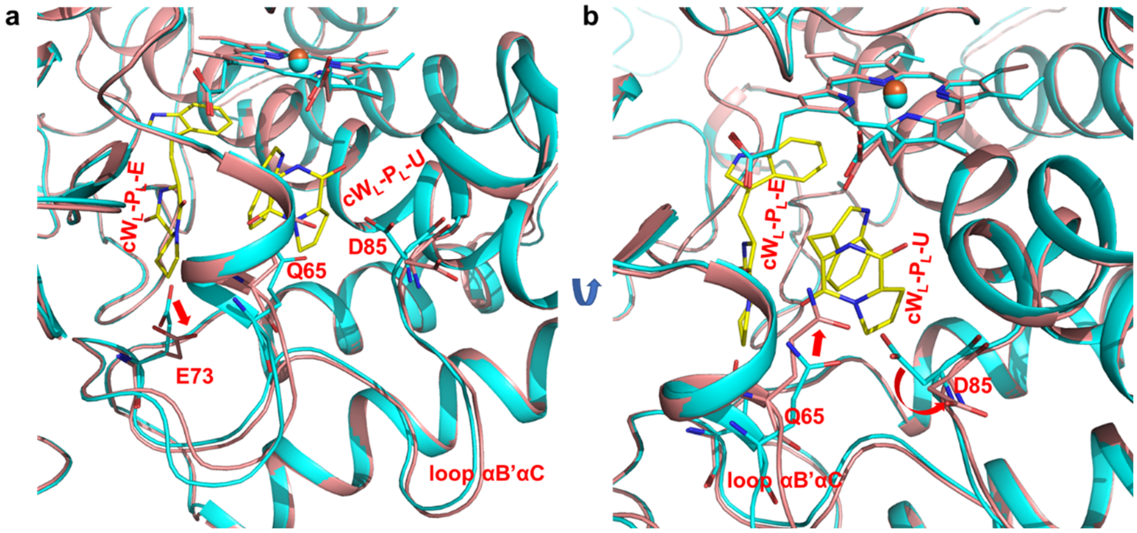


**Supplementary Fig. 12**. Superposition of Nas$_{F5053}$ without the substrates (cyan) and with the substrates (orange). (a) The two substrate molecules (cW$_L$-P$_L$-E and cW$_L$-P$_L$-U) are colored yellow. Oxygen and nitrogen atoms are colored red and blue, respectively. To accommodate substrates, the side-chains of D85 and E73 move away from the binding site, while the side-chain of Q65 shifts towards cW$_L$-P$_L$-U. Those movements are highlighted with red arrows. (b) As in Panel A, but rotated for a clearer view of Q65. Q65, D82 and E73 are all located in the long loop αB'-αC.

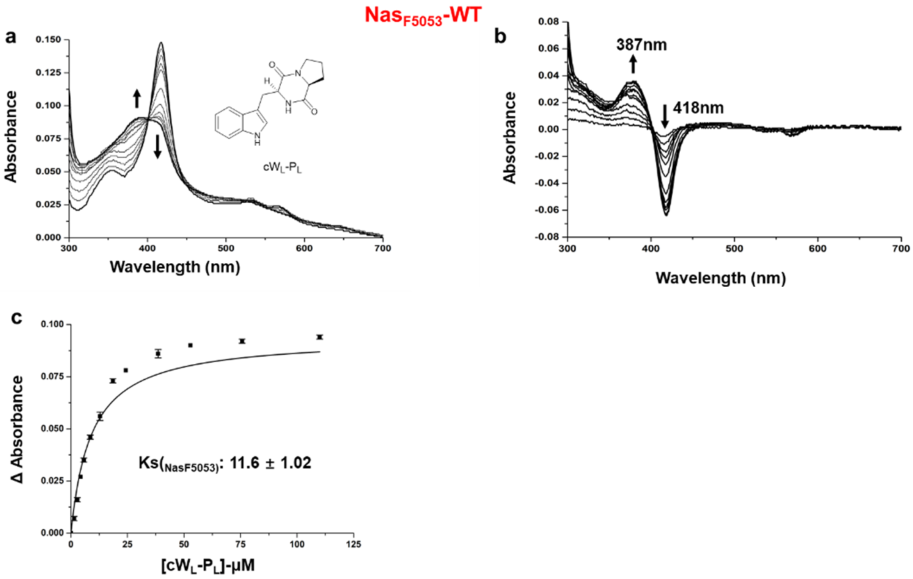
NasF5053-WT
a
b
c
Absorbance
Wavelength (nm)
cWL-PL
387nm
418nm
Δ Absorbance
Ks(NasF5053): 11.6 ± 1.02
[cWL-PL]-μM

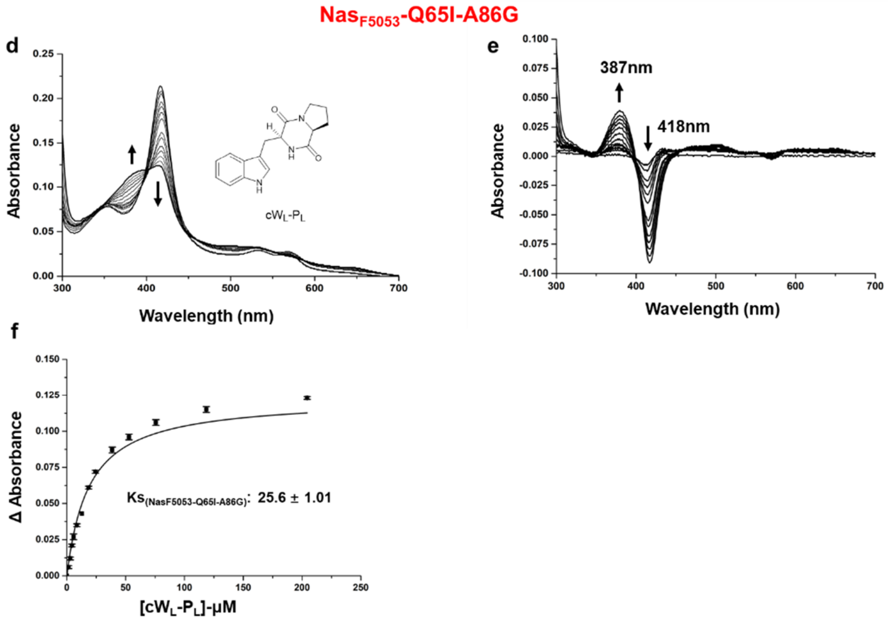
NasF5053-Q65I-A86G
d
e
f
Absorbance
Wavelength (nm)
cWL-PL
387nm
418nm
Δ Absorbance
Ks(NasF5053-Q65I-A86G): 25.6 ± 1.01
[cWL-PL]-μM

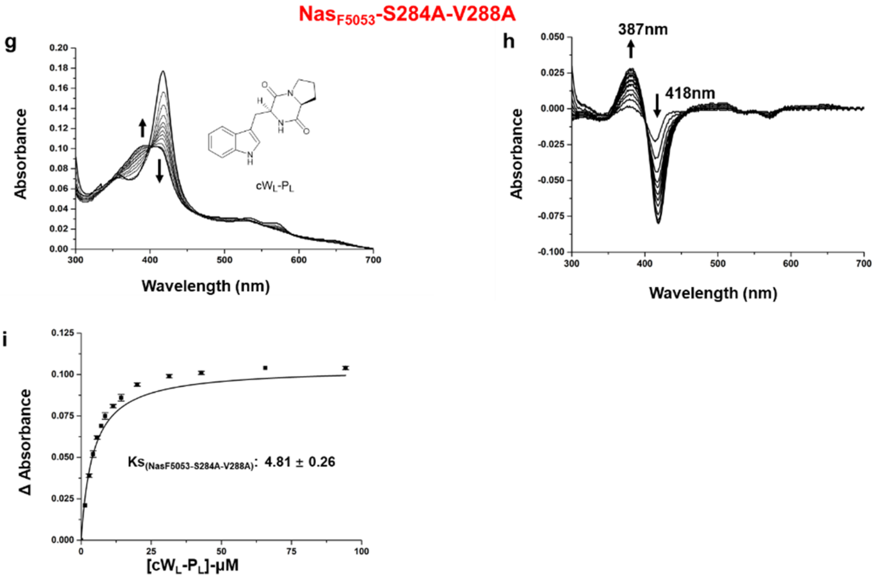


**Supplementary Fig. 13**. UV-Vis titration and determination of the binding constants of cW$_L$-P$_L$ to Nas$_{F5053}$ (a-c), Nas$_{F5053}$-Q65I-A86G (d-f) and Nas$_{F5053}$-S284A-V288A (g-i). The chemical structure of cW$_L$-P$_L$ is included beside UV absorption curve. Arrows indicate directions of change in spectra upon addition of cW$_L$-P$_L$. All these experiments are conducted independently in triplicate (n=3). The averaged values from triplicate were used to make plots. (a) Spectral titration of 2 μM Nas$_{F5053}$ with 0–110 μM cW$_L$-P$_L$. Spectra corresponding to 0 and 110 μM cW$_L$-P$_L$ are highlighted in bold black. (b) Difference spectra. (c) The fitting of titration points to a rectangular hyperbola curve for calculating the binding constant. (d) Spectral titration of 2 μM Nas$_{F5053}$-Q65I-A86G with 0–204 μM cW$_L$-P$_L$. Spectra corresponding to 0 and 204 μM cW$_L$-P$_L$ are highlighted in bold black. (e) Difference spectra for Nas$_{F5053}$-Q65I-A86G. (f) The fitting of titration points to a rectangular hyperbola curve for calculating the binding constant. (g). Spectral titration of 2 μM Nas$_{F5053}$-S284A-V288A with 0–95 μM cW$_L$-P$_L$. Spectra corresponding to 0 and 95 μM cW$_L$-P$_L$ are highlighted in bold black. (h) Difference spectra (i) The fitting of titration points to a rectangular hyperbola curve for calculating the binding constant.

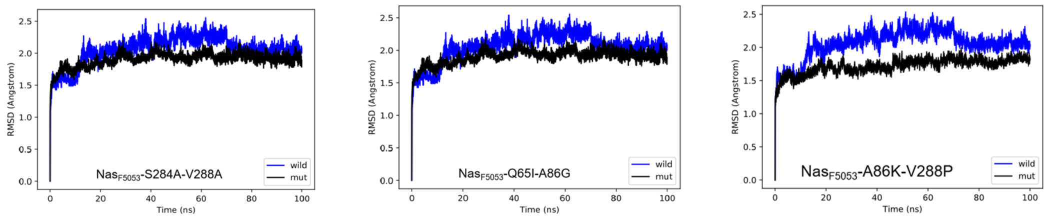


**Supplementary Fig. 14**. RMSD variation in molecular dynamics simulations.

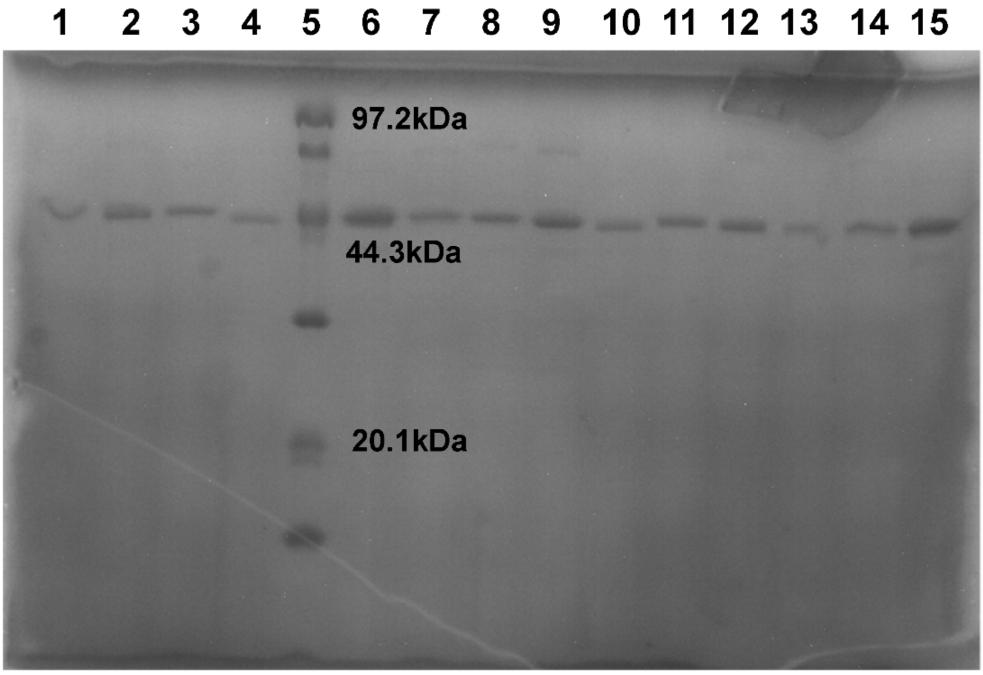


**Supplementary Fig. 15**. SDS-PAGE analysis of recombinant proteins. Lane 1: $Nas_{S1868}$, 44kDa; Lane 2: $Nas_{F5053}$, 44 kDa; Lane 3: NasbB, 44 kDa; Lane 4: $Nas_{F5053}$-Q65I, 44 kDa; Lane 5: Marker; Lane 6: $Nas_{F5053}$-A86G, 44 kDa; Lane 7: $Nas_{F5053}$-A86G-Q65I, 44 kDa; Lane 8: $Nas_{F5053}$-S284A, 44 kDa; Lane 9: $Nas_{F5053}$-V288A, 44 kDa; Lane 10: NascB-Q65I, 44 kDa; Lane 11: NascB-A86G, 44 kDa; Lane 12: NascB-Q65I-A86G, 44 kDa; Lane 13: NascB-S1868fragment-7, 44 kDa; Lane 14: NascB-Q65I-A86G-A284S-A288V, 44 kDa; Lane 15: $Nas_{F5053}$-A86K-V288P, 44 kDa.

## 2. Supplementary Tables

**Supplementary Table 1.** Product profiles and yields of the P450 reactions using $cW_L$-$P_L$ as substrate (NasB, NasbB, NascB, $Nas_{F5053}$ and AspB).

| Products & yields / P450s | NAS-B | NAS-C | ASP-A | Iso-NAS-B |
|---|---|---|---|---|
| NasB[a] | 93.5% | 0 | 6.5% | 0 |
| NasbB | 100% | 0 | 0 | 0 |
| NascB | 0 | 100% | 0 | 0 |
| $Nas_{F5053}$ | 8.2% | 47.4% | 44.4% | 0 |
| $Nas_{S1868}$ | 0 | 0 | 100% | 0 |
| AspB[a] | 0% | 1.5% | 98.1% | 0.4% |

[a]data were achieved from Li's paper[1].

**Supplementary Table 2.** NMR data of compound NAS-B in DMSO-$d_6$.

| *no.* | $\delta_H$ | $\delta_C$ | $^1$H-$^{13}$C HMBC | ROESY |
|---|---|---|---|---|
| 2 | 5.65, d,(3.2) | 85.0 | **7'** | **11,1-NH,12b,8'** |
| 3 | | 59.9 | | |
| 4 | | 134.7 | | |
| 5 | 6.73,d,(3.2) | 123.5 | 3,7,9 | |
| 6 | 6.54,m | 118.2 | 8 | |
| 7 | 6.97,m | 128.1 | 5, 9 | |
| 8 | 6.57,m | 109.4 | 4,6 | |
| 9 | | 148.2 | | |
| 11 | 4.66,m | 59.6 | 12,13 | **2**,12a |
| 12a | 3.10,m | 38.8 | 2,3,**7'** | |
| 12b | 2.33,m | | 3,13,7' | |
| 13 | | 166.1 | | |
| 15 | 4.30,s | 60.1 | 16,17 | |

| | | | |
|---|---|---|---|
| 16 | | 168.1 | |
| 17a | 2.11,m | 27.2 | |
| 17b | 1.35,m | | |
| 18a/b | 1.80,m,(2H) | 23.1 | |
| 19a/b | 3.21,m,(2H) | 44.7 | |
| 2’ | 7.15,s | 125.2 | 3’,4’,9’ |
| 3’ | | 109.2 | |
| 4’ | | 126.2 | |
| 5’ | 7.53,d,(8.4) | 119.3 | 3’,7’ |
| 6’ | 6.93,m | 118.0 | 3,4’,8’ |
| 7’ | | 135.7 | |
| 8’ | 7.28,s | 109.3 | 3,4’,6’ |
| 9’ | | 136.0 | |
| 11’ | 4.25,m | 55.3 | 3’,12’,13’ |
| 12’a | 3.16,m | 25.9 | |
| 12’b | 3.02,dd,(14.5,4.5) | | 2’,3’,11’,13’ |
| 13’ | | 165.6 | |
| 15’ | 4.02,m | 58.6 | 16’,17’ |
| 16’ | | 169.3 | |
| 17’a/b | 1.93,m,(2H) | 27.8 | |
| 18’a/b | 1.60,m,(2H) | 22.0 | |
| 19’a/b | 3.33,m,(2H) | 44.7 | |
| 1-NH | 6.69,s | | 3,4,9 |
| 1’-NH | 10.85,s | | 3’,4’,9’ |
| 10’-NH | 7.63,s | | 11’,15’,16’ |

**Supplementary Table 3.** NMR data of compound ASP-A in DMSO-$d_6$.

| ***no.*** | **$\delta_{H,}$mult (*J* in Hz)** | **$\delta_C$** | **$^1$H-$^{13}$C HMBC** |
|---|---|---|---|
| 2 | 7.28,d,(2.3) | 125.7 | 3,4,9 |
| 3 | | 109.7 | |
| 4 | | 126.1 | |
| 5 | 7.74,d,(8.4) | 119.7 | 3,7,9 |
| 6 | 7.14,dd,(8.7,1.4) | 115.2 | 4,8 |
| 7 | | 133.1 | |
| 8 | 7.44,d,(1.7) | 106.7 | 4,6 |
| 9 | | 136.1 | |
| 11 | 4.36,m | 55.3 | 3,12,13 |
| 12a | 3.31,m | 25.8 | 11 |
| 12b | 3.29,m | | |
| 13 | | 165.5 | |
| 15 | 4.10,m | 58.5 | 16,17 |
| 16 | | 169.1 | |
| 17a/b | 1.40,m(2H) | 27.7 | |
| 18a/b | 1.70,m(2H) | 21.9 | |
| 19a | 3.28,m | 44.6 | |
| 19b | 3.26,m | | |
| 2’ | 7.46,s | 128.3 | 3’,4’,9’,**7** |
| 3’ | | 111.0 | |
| 4’ | | 128.3 | |
| 5’ | 7.68,d,(7.8) | 119.4 | 3’,7’,9’ |
| 6’ | 7.47,d,(8.2) | 110.1 | 4’,8’ |
| 7’ | 7.17,m | 122.0 | 5’,9’ |
| 8’ | 7.10,m | 119.4 | 4’,6’ |
| 9’ | | 135.6 | |
| 11’ | 4.40,m | 55.2 | 3’,12’,13’ |

| | | | |
|---|---|---|---|
| 12’a | 3.16,d,(5.6) | 25.9 | 2’,3’,4’,11’,13’ |
| 12’b | 3.13,d,(5.6) | | 2’,3’,4’,11’,13’ |
| 13’ | | 165.4 | |
| 15’ | 4.08,m | 58.5 | 16’,17’ |
| 16’ | | 169.0 | |
| 17’ | 2.00,m,(2H) | 27.7 | |
| 18' | 1.61,m,(2H) | 21.8 | |
| 19’a | 3.42,m | 44.6 | |
| 19’b | 3.38,m | | |
| 1-NH | 11.05,d,(2.2) | | 3,4,9 |
| 10-NH | 7.85,s | | 11,15,16 |
| 10’-NH | 7.97,s | | 11’,15’,16’ |

**Supplementary Table 4.** NMR data of compound NAS-E in DMSO-$d_6$.

| ***no.*** | $\delta_H$ | $\delta_C$ | **$^1$H-$^{13}$C HMBC** | **ROESY** |
|---|---|---|---|---|
| 2 | 5.56,s | 81.4 | 4,9,7’ | **1-NH,6’,8’** |
| 3 | | 58.8 | | |
| 4 | | 132.4 | | |
| 5 | 7.16,d,(8.4) | 124.0 | 3,7,9 | |
| 6 | 6.66,m | 109.5 | 8 | |
| 7 | 6.99,dd,(7.6,1.1) | 128.2 | 5,9 | |
| 8 | 6.64,d,(2.6) | 118.1 | 4,6 | |
| 9 | | 149.4 | | |
| 11 | 4.17,m | 60.2 | 13 | |
| 12a | 3.06,m | 40.0 | 2,4 | |
| 12b | 2.66,m | | 3,4,13,7’ | |
| 13 | | 165.3 | | |
| 15 | 4.30,s | 59.6 | 17,18 | |
| 16 | | 166.1 | | |

| | | | |
|---|---|---|---|
| 17a | 2.14,m | 27.4 | |
| 17b | 1.98,m | | |
| 18a | 1.94,m | 22.6 | |
| 18b | 1.81,m | | |
| 19a | 3.17,m | 44.6 | |
| 19b | 3.26,m | | |
| 2’ | 7.14,d,(2.6) | 124.8 | 3’,4’,9’ |
| 3’ | | 109.2 | |
| 4’ | | 126.1 | |
| 5’ | 7.50,d,(8.4) | 119.0 | 3’,9’ |
| 6’ | 7.07,dd,(8.4,1.5) | 116.4 | 4’,8’,3, |
| 7’ | | 136.8 | |
| 8’ | 7.23,s | 108.0 | 3,4’,6’ |
| 9’ | | 136.0 | |
| 11’ | 4.26,m | 55.2 | 3’,12’,13’ |
| 12’a | 3.22,m | 25.7 | 2’,3’,4’,11’,13’ |
| 12’b | 3.03,m | | |
| 13’ | | 165.5 | |
| 15’ | 4.05,m | 58.4 | 16’,17’ |
| 16’ | | 169.1 | |
| 17’a | 1.89,m | 27.6 | |
| 17’b | 1.41,m | | 15’,16’ |
| 1-NH | 6.62,s | | 3,4 |
| 1’-NH | 10.77,d,(2.1) | | 3’,4’,9’ |
| 10’-NH | 7.65,s | | 13’,15’ |

**Supplementary Table 5.** X-ray data collection and structure refinement statistics for

306 $Nas_{F5053}$ and its mutants.

307

| | Substrate-bound $Nas_{F5053}$ | Ligand-free $Nas_{F5053}$ | Substrate-bound $Nas_{F5053}$-Q65I/A86G | Substrate-bound $Nas_{F5053}$-S84A/V288A |
|---|---|---|---|---|
| **Data collection** | | | | |
| Space group | P 2 $2_1$ $2_1$ | P $2_1$ | P 2 $2_1$ $2_1$ | P 2 $2_1$ $2_1$ |
| Cell dimensions | | | | |
| *a*, *b*, *c* (Å) | 42.31, 91.84, 93.60 | 42.21, 91.33, 98.26 | 42.23, 91.31, 93.81 | 42.24, 91.21, 93.46 |
| α, β, γ (°) | 90.00, 90.00, 90.00 | 90.00, 96.41, 90.00 | 90.00, 90.00, 90.00 | 90.00, 90.00, 90.00 |
| Wavelength (Å) | 0.954 | 0.954 | 0.954 | 0.954 |
| Resolution (Å) | 46.80-1.60 (1.63-1.60) | 43.06-1.70 (1.76-1.70) | 38.51-1.47 (1.52-1.47) | 46.73-1.68 (1.74-1.68) |
| CC1/2 | 0.99 (0.71) | 1.00 (0.79) | 1.00 (0.68) | 1.00 (0.74) |
| $R_{merge}$ | 0.085 (1.177) | 0.035 (0.326) | 0.026 (0.329) | 0.039 (0.335) |
| Average $I / \sigma (I)$ | 11.8 (1.7) | 9.1 (2.2) | 14.7 (2.2) | 12.5 (2.1) |
| Completeness (%) | 99.9 (99.7) | 99.7 (99.1) | 98.4 (95.6) | 98.1 (96.4) |
| Multiplicity | 6.7 | 6.4 | 6.8 | 6.7 |
| **Refinement** | | | | |
| Resolution (Å) | 46.80-1.60 (1.63-1.60) | 43.06-1.70 (1.76-1.70) | 38.51-1.47 (1.52-1.47) | 46.73-1.68 (1.74-1.68) |
| No. unique reflections | 48951 (2415) | 81166 (8040) | 61852 (5917) | 41354 (3988) |
| $R_{work}$ / $R_{free}$ (%) | 16.9/20.3 | 19.3/21.9 | 15.5/18.0 | 16.9/24.7 |
| No. atoms | | | | |
| Protein | 3068 | 6133 | 3050 | 3037 |

| | | | | |
|---|---|---|---|---|
| Ligand/ion | 5 | 13 | 8 | 2 |
| Water | 351 | 812 | 423 | 342 |
| *B*-factors (Å$^2$) | | | | |
| Protein | 25.9 | 23.1 | 18.9 | 19.0 |
| Ligand/ion | 18.3 | 16.5 | 14.7 | 12.5 |
| Water | 35.9 | 32.5 | 30.1 | 26.0 |
| R.m.s deviations | | | | |
| Bond lengths (Å) | 0.005 | 0.023 | 0.009 | 0.010 |
| Bond angles (°) | 0.81 | 1.79 | 1.09 | 1.11 |

*Values in parentheses are for the highest-resolution shell.

$R_{merge} = \sum_{hkl}\sum_{j}|I_{hkl,j} - <I_{hkl}>|/(\sum_{hkl}\sum_{j}I_{hkl,j})$

$R_{work}$ / $R_{free}$ = $\sum_{hkl}\left|F_{hkl}^{obs} - F_{hkl}^{calc}\right|/(\sum_{hkl}F_{hkl}^{obs})$; $R_{free}$ was calculated using randomly chosen 5-10 % fraction of data that was excluded from refinement.

**Supplementary Table 6.** Bacterial strains and plasmids.

| Strains | Description | Source |
|---|---|---|
| DH5α | Host for general cloning | Invitrogen |
| BL21 (DE3) | Host for protein expression | Invitrogen |
| GB05-dir-T7 | Host for protein expression | This study |
| *M. smegmatis* mc$^2$ 155 | Host for protein expression | From Prof. Jiaoyu Deng |
| **Plasmids** | | |
| pET28a | Protein expression vector in *E. coli* | Novagen |
| pET21a | Protein expression vector in *E. coli* | Novagen |
| pMF406 | Protein expression vector in *M. smegmatis* mc$^2$ 155 | From Prof. Xiaoyong Fan[2] |

| | | |
|---|---|---|
| pMV261 | Protein expression vector in *M. smegmatis* mc$^2$ 155 | From Prof Jiaoyu Deng |
| pMV261-ACE | pMV261 derivative containing pACE | This study |
| pSrtA9 | | [6] |
| pMS1 | Protein expression vector in *M. smegmatis* mc$^2$ 155 | This study |
| pSJTU1 | pET28a derivative for $\text{Nas}_{F5053}$ expression in *E. coli* | This study |
| pSJTU2 | pMS1 derivative for NasbB expression in *M. smegmatis* mc$^2$ 155 | This study |
| pSJTU3 | pMS1 derivative for $\text{Nas}_{S1868}$ expression in *M. smegmatis* mc$^2$ 155 | This study |
| pSJTU4 | pET21a derivative, containing $\text{Nas}_{F5053}$ gene | This study |
| pWHU2487 | pRSF-Duet derivative for Trx-Fd and MBP-FdR *co*-expression in *E. coli* | [3] |
| pWHU2485 | pET28a derivative for NascB expression in *E. coli* | [3] |

**Supplementary Table 7.** Primers used in this study.

| **Name** | **Sequence (**5’ to 3’) |
|---|---|
| F5053-F | tggtgccgcgcggcagccatatgATGACCACCACCGCAACCCTGACC |
| F5053-R | ttgtcgacggagctcgaattcTTACCATGTTGCCGGAATGGCGCGC |
| F5053-overlap-R | GCTGCGGCGTAGCGTGTTGCCAGCCACAGATGGTCAC |
| F5053-overlap-F | ACACGCTACGCCGCAGCCGTGGAACTGCTGGAAG |
| F5053-A86G-F | CGGTGACGGTATTGCAATG |
| F5053-A86G-R | CATTGCAATACCGTCACCG |

| | |
|---|---|
| F5053-Q65I-F | GAAGCCGCAATTGCCAGTGGT |
| F5053-Q65I-R | ACCACTGGCAATTGCGGCTTC |
| F5053-I87V-F | GGTGACGCCGTGGCAATGCTG |
| F5053-I87V-R | CAGCATTGCCACGGCGTCACC |
| F5053-G84A-F | GTACACGCGCCGACGCCATTG |
| F5053-G84A-R | CAATGGCGTCGGCGCGTGTAC |
| F5053-Xray-For | GCCGGAAACCGGCTCTACCCTGACCTACCCGTTTCATGAC |
| F5053-Xray-Rev | GCAAAGCACCGGGGCTTACCATGTTGCCGGAATGGCG |
| $Nas_{F5053}$-S284A-V288A-For | cagcgcaAAACGCATGGCCACCGAA |
| $Nas_{F5053}$-S284A-V288A-Rev | cctgttgcCAGAGGGGTATAACGCAGC |
| nascB-A86G-F | TACACGTGGTGACGGTATCGCAATG |
| nascB-A86G-R | GTAACATTGCGATACCGTCACCACG |
| nascB-Q65I-F | TGAAGCCGCAATTGCAAGCGGTGC |
| nascB-Q65I-F | CGGGGCACCGCTTGCAATTGCG |
| nascB-P40H-F | TACCGGCGATCATCTGTGGCTGG |
| nascB-P40H-R | TAACCAGCCACAGATGATCGCCG |
| nascB-V44A-F | CTGTGGCTGGCAACCCGCTATGC |
| nascB-V44A-R | GTGGCATAGCGGGTTGCCAGCCA |
| nascB-T49A-F | ACCCGCTATGCCGCCGCCGTTAA |
| nascB-T49A-R | AGTTTAACGGCGGCGGCATAGCG |
| nascB-K52E-F | CACCGCCGTTGAACTGCTGGAAG |
| nascB-K52E-R | TGTCTTCCAGCAGTTCAACGGCGGT |
| nascB-S57F-F | GCTGGAAGACTTCCGCTTCAGCA |
| nascB-S57F-R | CACTGCTGAAGCGGAAGTCTTCC |
| nascB-G84A-F | GGTACACGTGCAGACGCAATCGC |
| nascB-G84A-R | ATTGCGATTGCGTCTGCACGTGT |
| nascB-I87V-F | GGTGACGCAGTGGCAATGTTA |

| | |
|---|---|
| nascB-I87V-R | GTAACATTGCCACTGCGTCAC |
| nascB-For | tggtgccgcgcggcagccatatgATGACCACCACCGCAACCCTG |
| nascB-Rev | ttgtcgacggagctcgaattcCCAGGTGGCAGGCAGTGCGC |
| nascB-fragment1-R | GCTCATCAGGGTTTCGGCCAGATCATTAATCCAGCCC |
| nascB- fragment1-F | GCCGAAACCCTGATGAGCGCACTGGCCAGCCGTGAAG |
| nascB- fragment2-R | CTGCTCAGAGGTTCCACAAAATCGGCTG |
| nascB- fragment2-F | GATCTGGCAGCCGATTTTGTGGAACCTC |
| nascB- fragment3-R | GCGCAGGCCTGTGTCTGCACAGTGTGCCAGCAGATCGC |
| nascB-fragment3-F | GCAGACACAGGCCTGCGCTTTTGCGGCGTGACCCATG |
| nascB-fragment4-R | GCTCCAGAAAAAATTCGTGCATCTGGGTGAAGGCGTGAACC |
| nascB-fragment4-F | ACGAATTTTTTCTGGAGCATGCACGTCGCTTAGCAGGCACCCC |
| nascB-fragment5-R | CATCGCTTAACGGACCATGATCCACCGGTGCCTCGGCAATCAGT<br>T |
| nascB-fragment5-F | ATGGTCCGTTAAGCGATGAAGCCCTGGCCGAAGCAGGCAGCCT<br>GC |
| nascB-fragment6-R | CAGCTCCTGCACGGCATCCGGGTGACGCAGCAGTGTCAGCA |
| nascB-fragment6-F | CACCCGGATGCCGTGCAGGAGCTGCATGCACATCCGGAACGCG<br>T |
| nascB-fragment7-R | CATGCGTTTCACGCTGCCTGTGCTCAGCGGTGTATAGCGC |
| nascB-fragment7-F | GGCAGCGTGAAACGCATGGCAACAGAGGACCTGGAGATTGA |
| nascB-fragment8-R | CCTCCAGGCTAACCATCACCACCTCACCCACTTTAAT |
| nascB-fragment8-F | GTGATGGTTAGCCTGGAGGCCGTTAACCATGACCCGG |
| nascB-H280Y-F | ACTGCTGCGCTATACACCGCTGGCA |
| nascB-H280Y-R | TGCCAGCGGTGTATAGCGCAGCAGT |
| nascB-A288V-F | ACAGGCGCCGTGAAACGCATGGCA |
| nascB-A288V-R | TGCCATGCGTTTCACGGCGCCTGT |
| nascB-A287S-F | CTGGCAACAGGCAGCGCAAAACGCA |
| nascB-A287S-R | TGCGTTTTGCGCTGCCTGTTGCCAG |
| nascB-A284S-F | CATACACCGCTGAGCACAGGCGCCG |

| | |
|---|---|
| nascB-A284S-R | CGGCGCCTGTGCTCAGCGGTGTATG |
| nascB-Y280H-F | GAACTGCTGCGCCATACACCGCTG |
| nascB-Y280H-R | GCTCAGCGGTGTATGGCGCAGCAG |
| nascB-V288A-F | CACAGGCAGCGCAAAACGCATGG |
| nascB-V288A-R | TGCCATGCGTTTTGCGCTGCCTG |
| nascB-S287A-F | GAGCACAGGCGCCGTGAAACGCAT |
| nascB-S287A-R | CCATGCGTTTCACGGCGCCTGTGC |
| nascB-S284A-F | TACACCGCTGGCAACAGGCAGCG |
| nascB-S284A-R | CACGCTGCCTGTTGCCAGCGGTG |
| nascB-I298L-F | GGACCTGGAGCTGGATGGTGTTC |
| nascB-I298L-R | ACGAACACCATCCAGCTCCAGGT |
| $\text{Nas}_{\text{F5053}}$-S284A-F | ATACCCCTCTGGCAACAGGCAGC |
| $\text{Nas}_{\text{F5053}}$-S284A-R | TCACGCTGCCTGTTGCCAGAGGGGT |
| $\text{Nas}_{\text{F5053}}$-V288A-F | GCACAGGCAGCGCAAAACGCATG |
| $\text{Nas}_{\text{F5053}}$-V288A-R | GGCCATGCGTTTTGCGCTGCCTGT |
| $\text{Nas}_{\text{F5053}}$-SM-NNK-F | CGCANNKGCCAGTGGTGCACCGCGTCAGGAACCGGTGGAATTA<br>CGTGCCCCGGGTACACGCGGTGACNNKAT |
| $\text{Nas}_{\text{F5053}}$-SM-NNK-R | TGGCCATGCGTTTMNNGCTGCCTGTMNNCAGAGGGGTATAACG<br>CAG |
| Ami-for | TTTTCTAGAGAAGTGACGCGGTCTCAAGCG |
| Ami-rev | TTTGGATCCGAAAACTACCTCGGGCATGTGGAC |
| tev-for | GATCGCACCATCACCATCACCACGGTGGTTCGGGCGAGAACCT<br>GTACTTCCAGGGATCCCATATGG |
| tev-rev | CGTGGTAGTGGTAGTGGTGCCACCAAGCCCGCTCTTGGACATG<br>AAGGTCCCTAGGGTATACCTTAA |

## 3. Supplementary Methods

### 3.1. General materials and methods

*Escherichia coli* and *Mycobacterium* strains were cultivated and manipulated according to standard methods[2,4]. *Mycobacterium smegmatis* mc$^2$ 155 was cultivated either on Luria-Bertani (LB) media agar plates or in Lemoco liquid media (5 g peptone, 5 g beef extract, 5 g NaCl, 0.1 % Tween 80 in 1 L tap water). Strains and plasmids used in this study are listed in **Supplementary Table 6**. Primer synthesis and DNA sequencing were performed at Genewiz Biotech Co., Ltd. (China). All the primers used in this study are all listed in **Supplementary Table 7**. Restriction enzymes and DNA polymerases (Taq and PrimeSTAR) were purchased from Takara Biotechnology Co., Ltd. (China). Discovery Studio was used to analysis the interactions between ligands and the enzymes. All chemicals and reagents were purchased from Santa Cruz Biotechnology, Inc. (USA) or Shanghai Sangon Biotech (China) Co., Ltd., unless noted otherwise, and all the chemical structures were drawn with ChemBioDraw. HPLC analysis was carried out on an SHIMADZU LC-30A UPLC system. UPLC-MS analysis was carried out on a SHIMADZU LC-30A system connected to a single quadrupole mass spectrometer MS2020 (ESI). ESI-high resolution MS (ESI-HRMS) analysis was carried out on ESI-LTQ Orbitrap (ThermoFisher Scientific Inc.). NMR analysis was carried out on a Bruker DRX-400 NMR spectrometer (Bruker Co. Ltd., Germany).

### 3.2. Cloning, expression and purification of P450s and associated proteins

The DNA sequences of $Nas_{F5053}$, NasbB and $Nas_{S1868}$ were optimized based on *E. coli* codon bias and synthesized (GENEWIZ Co. Ltd., see below). The DNA fragment of $Nas_{F5053}$ was released by *Nde*I-*Xho*I digestion and cloned into the same sites of pET28a to generate pSJTU1. The resulting plasmid was transferred into *E. coli* BL21 (DE3) for protein expression. After expression in 500 mL culture at 16 °C and 220 rpm for 20 h following 100 μM isopropyl-β-D-thiogalactopyranoside (IPTG) induction (IPTG, 0.4 mM δ-aminolevulinic acid (ALA) and 0.2 mM $(NH_4)_2Fe(SO_4)_2$ were added when $OD_{600}$ reached ~0.6), cells were harvested by centrifugation at 5,000 rpm at 4 °C. The cell pellet was resuspended in 20 mL lysis buffer (25 mM HEPES, pH 7.5, 300 mM

NaCl, 5 mM imidazole, 10% glycerol) and lysed by ultra-sonication. The insoluble debris was removed by centrifugation at 12,000 rpm, 4 °C for 1 h. The protein supernatant was then incubated with 1 mL Ni-NTA sepharose for 1.5 h with slow, constant rotation at 4 °C. Subsequently, the protein-resin mixtures were loaded into a gravity flow column, and proteins were eluted with increasing concentrations of imidazole (25 mM, 50 mM, 100 mM, 300 mM) in Buffer A (25 mM HEPES, pH 7.5, 300 mM NaCl, 10% glycerol). Purified proteins were then loaded into PD-10 desalting columns and desalted using buffer B (25 mM HEPES, pH 7.5, 50 mM NaCl, 10% glycerol) and concentrated by centrifugation using an Amicon Ultra-4 (GE Healthcare). The purified proteins were evaluated by 12% acrylamide SDS-PAGE (**Supplementary Fig. 15**).

Because NasbB and $Nas_{S1868}$ could not be expressed as soluble proteins in *E. coli*, we employed the *Mycobacterium* system for their expression. Firstly, a new plasmid named pMS1 was developed by using the following method: the *pACE* gene was amplified from the pMF406 vector with the primer pair Ami-for/Ami-rev and cloned into the *Xba* I/ *Bam*H I sites of the pMV261 vector to generate the plasmid pMV261-ACE. Further, a gene fragment, obtained by annealing with the primers pair tev-for/tev-rev was cloned into the *Bam*H I/ *Eco*R I sites of pMV261-ACE, to yield the pMS1. Secondly, the DNA fragments of these two P450s were cloned into the site between *Nde* I and *Eco*R I of pMS1, to generate pSJTU2 and pSJTU3. These expression plasmids were then electro-transformed into *Mycobacterium smegmatis* mc $^{2}$155 for overexpression of N-terminal 6× His-tagged fusion proteins. In 0.8 L of liquid culture, the cells were grown at 30 °C in Lemoco media with 50 µg $mL^{-1}$ kanamycin to an $OD_{600}$ of 1.0-1.5. The cells were then induced by 30 mM acetamide, 0.4 mM *δ*-aminolevulinic acid (ALA) and 0.2 mM $(NH_4)_2Fe(SO_4)_2$ for 20 h at 30 °C. The cells were then harvested and the subsequent steps were the same as for *E. coli* expression. Protein concentration was determined by the Bradford method using a BSA calibration curve. The purified proteins were stored at -80 °C and used for in vitro assays.

Sequences of synthetic P450s (restriction enzyme recognition sites are underlined):

*nasbB*:

CATATGATTCGTCCGCAGCCGCATCGCAGTCCGGTTGACCCGTATACCAAAGAAT
GCCGTACCGTGACCACCGCACCGGTTCCGCTGACCTTTCCTTTCCACGACTGG
AGTCAAGAGCTGAGTCCGCATCATGAACGTCTGCGCGAAGCAGATGCCCCGGT
GTGTCCGGTGGTGAGCGAATATACCGGCGATCGCCTGTGGCTGGTGACACGCT
ACGCCACCGCAAAACGCCTGCTGGAGGATCGCCGTTTTAGCAGTACCGCCGCA
ATGGCACCTGGTGCACCGCGTCAGGAACCGGTGGAATTACGCGCACCGGGTAC
CACCGGTGACGGTGTGAGCGTTCTGCGCGAGGCAGGCCTGCGTACAGTGTTCA
CCGAAGGTTTAGGTCCGCGCGCAGCCCGTCGTCATGGTAAATGGCTGCGCGAT
CGTGCAGATACCTTACTGCGCGATGTTGCCGAGTGCGAAGGCCCGGTGGATCT
GGCAGCCGATTTTGCACAGCCGCTGGCCGTGGCAATGACAAGTCGCGTGCTGC
TGGGTGAACTGAGCACCGAAGAAGCCGCACTGTTACGTGATCGTACCGACCTG
GCCCTGCAGTTTTGTGGTGCAACCGCCGAAGAACAGCGCGGCGGTCTGATTGA
TATTCATCGCTTTTTTACCGCCCATGCCCGCCGCTTAGCAGATGGTCCGGGTGAC
CACCTGCTGAAACGTCTGGCCGAAGCCCCGGCAGAAAATGGCCCGCTGGGTGA
TGCCGCCCTGAGTGAAATTGCCGCCCTGCTGCTGATTGCAGGCTTCCCGACCA
GTAGCGGCTTCCTGTGCGGTGCCCTGATCACACTGCTGCGCCATCCGGAAGCC
GTTGGTCGTCTGCGTCGCGATCCGGAACTGATTCCTGACGCCGTGGAAGAACT
GCTGCGTCATACCCCGCTGAGCACCGGTGCCGCAAAACGTATGGCCACCGAAG
ATGCCGATATTGACGGCGTTCGCATTCGCCGCGGTGAAGTGGCCATGGTTAGCC
TGGAAGCCGCCAACCATGATCCTGACGCCTTCGATGATCCGGACAGTTTTCGTC
CGGAACGCCAGGGTCCGGGTCATCTGGGTTTTGGCCATGGCCCGAATTTTTGC
CCGGGTAATCGCCTGGCACGCTGTCTGATCGATGCCATGGTTCGTGCCGTTGCA
CGCCGTCCGGGTTTACACCTGACAGTGGGCCCGGAGGAGATCCGCTGGCATGA
AGGTCTGTTTTTCCGCCGTCCGAAAGCCATCCCGGCAAGCTGGTAACTCGAG

$nas_{S1868}$:

CATATGACAACCACCGCCACCCTGACCTATCCGTTTCACGATTGGAGCCAGGAG
CTGAGTCCGCGTTACGCCCAGCTGCGTGCCAGTGATGCCCCGGTTTGCCCGGT
TGTGAGTGAAGGTACCGGCGATCATCTGTGGCTGGCAACCCGCTATGCCGCAG
CCGTTGAACTGCTGGAAGACCCTCGTCTGAGCAGCGAAGCCGCAATTGCAAGC

GGTGCACCTCGTCAGGAGCCGGTTGAACTGCGTGCACCTGGTACCCGTGCAGATGGTGTTGCAATGCTGCGTGAAGCCGGCCTGCGCAGTGTTCTGGCAGACGGTCTGGGTCCGCGCGCAGTTCGTCGCCATCAGGGCTGGATCAATGACTTAGCCGAAACCCTGATGAGCGCATTAGCAAGTCGCGAGGGCACCTTTGACCTGGCCGCCGATTTTGTGGAACCGCTGAGTAGTGCCCTGGTGAGCCGTACCCTGCTGGGCGAACTGAGCGCAGATGAACGCGATCTGCTGGCCCATTGTGCCGATACCGGTCTGCGCTTCTGCGGTGTTACCCACGAAGAGCAGGTGCATGCCTTCACCCAGATGCATGAGTTCTTCCTGGAGCATGCCCGTCGTCTGGCAGGTACACCGGGTGAACACCTGCTGAAACTGATCGCCGAAGCCCCGGTTGATCACGGCCCGTTAAGTGATGAAGCCCTGGCCGAAGCCGGTAGTCTGCTGGTGGTGGCAGGTTTTCCGACCAGCAGCGGCTTTCTGTGCGGTGCACTGCTGACCTTACTGCGCCATCCGGATGCCGTTCAGGAGCTGCACGCCCATCCGGAACGCGTTCCTAGTGCCGTTGAGGAACTGCTGCGCTATACCCCGCTGAGCACCGGTAGCGTGAAACGCATGGCAACCGAAGATCTGGAAATCGACGGCGTGCGCATCAAAGTGGGTGAAGTGGTGATGGTGAGCCTGGAAGCCGTGAATCATGACCCGGATGCCTTCGAAGATCCGGACGTGTTTCGTCCGGGTCGCGAAGGCCCTATGCACTTTGGTTTTGGCCGCGGTCGCCATTTTTGTCCGGGTAACCGCCTGGCCCGCTGTGTGATTGAAGCCACCGTTCGTGCAGTTGCACGTCGCCCGGGTCTGCGTTTAGCAGTTGCCCCGGAGGAAATCAGCTGGCATGAAGGCCTGTTCTTTCGTCGCCCTCGTGCCATCCCGGCCACATGGTAAGCTT

*nas*$_{F5053}$:

CATATGACCACCACCGCAACCCTGACCTACCCGTTTCATGACTGGAGCCAGGAACTGAGCCCGCGTTATGCCCAGCTGCGTGCAAGCGATGCACCGGTGTGTCCGGTGGTGAGTGAAGGTACCGGTGACCCGCTGTGGCTGGTGACACGCTACGCCACCGCCGTGAAACTGCTGGAAGATAGCCGCTTTAGCAGTGAAGCCGCACAGGCCAGTGGTGCACCGCGTCAGGAACCGGTGGAATTACGTGCCCCGGGTACACGCGGTGACGCCATTGCAATGCTGCGCGAGGCCGGTCTGCGTAGCGTTCTGGCCGACGGCTTAGGTCCTCGTGCAGTGCGCCGTCATCAGGGTTGGATCAACGACCTGGCCGAAACCCTGATGAGCGAATTAGCAAGCCGTGAAGGCACCTTTGACCTGGCCGCAGATTTTGTGGAACCGCTGAGTAGCGCCCTGGTGAGTCGTACACTGCTGGGCGAGCTGAGCGCAGACGAACGCGATCTGCTGGCACATTGCGCCGATACCGGTCTGCGCT

TTTGCGGTGTGACACATGAAGAACAGGTGCACGCCTTCACCCAGATGCATGAGT
TCTTCCTGGAGCATGCACGTCGTCTGGCAGGTACCCCGGGTGAGCACCTGTTAA
AACTGATTGCCGAGGCCCCGGTTGATCAGGGTCCGCTGAGCGATGAGGCCCTG
GCAGAAGCAGGTAGCCTGCTGGTTGTGGCAGGCTTCCCGACCAGCAGCGGCTT
TCTGTGCGGTGCACTGCTGACCCTGCTGCGCCATCCGGATGCCGTGCAAGAAC
TGCATGCCCACCCGGAACGTGTGCCTAGCGCAGTGGAAGAGCTGCTGCGTTATA
CCCCTCTGAGCACAGGCAGCGTGAAACGCATGGCCACCGAAGACCTGGAAATT
GACGGCGTGCGCATCAAAGCCGGTGAAGTGGTTATGGTGAGCCTGGAAGCAGT
GAACCATGATCCGGATGCCTTTGAGGATCCGGATGTTTTTCGCCCGGGTCGCGA
AGGTCCGATGCACTTTGGTTTTGGTCGTGGCCGTCATTTCTGTCCGGGCAATCG
CCTGGCACGTTGCGTGATTGAAGCAACCGTGCGTGCAGTTGCACGTCGTCCGG
GTCTGCGTCTGGCAGTGGCACCGGAAGAGATCAGCTGGCACGAGGGTCTGTTT
TTTCGCCGTCCGCGCGCCATTCCGGCAACATGGTAAGCTTGAATTC

### 3.3. P450 enzyme assays

The activities of wild-type and mutant P450s were assayed in HEPES buffer (50 mM HEPES, 100 mM NaCl, pH 7.5) containing 0.1 μM purified P450s, 1 mM $cW_L$-$P_L$, 1 μM spinach ferredoxin (Fd), 1 μM spinach ferredoxin reductase (FdR), 2 mM $NADP^+$, 2 mM glucose and 2 mM glucose dehydrogenase (GDH). Expression and purification of Fd, FdR, GDH were described previously[3]. The reaction was incubated at 4 °C. After 24 h, two times the volume of ethyl acetate was added to quench the reaction, followed by sonication for 5 minutes. After the separation of aqueous and organic phases, the ethyl acetate phase was transferred to a rotavapor to dry, which was re-dissolved in HPLC-graded methanol. The resultant solution was filtered through a 0.45 μM membrane and subjected to analysis by UPLC-MS. A Diamonsil (C18, 2 μm, 2.1× 50 mm, Shim-pack GIST) was used with a flow rate at 0.3 mL $min^{-1}$ and a PDA detector over a 23 min gradient program with water (eluent A) and methanol (eluent B): T = 0 min, 40% B; T = 10 min, 40% B; T = 15 min, 70% B; T = 18 min, 40% B; T= 23 min, 40% B.

### 3.4. Scaled biocatalytic reaction and purification of NAS-B, ASP-A and NAS-E

Based on the whole-cell catalysis system we developed previously[3], genes of $Nas_{F5053}$ or its mutants, together with the plasmid pWHU2487 expressing Fd and FdR, were co-transferred into *E. coli* GB05-dir-T7. The resulting bacteria were inoculated in LB media (5 L) and grown to an $OD_{600}$ of 0.8-1.0 at 37 °C. After this, the cells were shifted to 18 °C, 220 rpm and supplemented with 100 μM isopropyl-*β*-D-thiogalactopyranoside (IPTG), 0.4 mM δ-aminolevulinic acid (ALA) and 0.2 mM $(NH_4)_2Fe(SO_4)_2 \cdot 6H_2O$, for expression for additional 20 h. The cells were harvested by centrifugation at 5,000 rpm at 4 °C and resuspended in 200 mL M9 medium. Then, 100 mM $cW_L$-$P_L$ (1 mL) was added in M9 media. After 48 h incubation at 18 °C, the reaction mixture was extracted with 400 mL ethyl acetate three times. The organic phase was transferred and dried by vacuum at a low temperature. Metabolites were subsequently redissolved by methanol and filtrated by a 0.45 μm membrane to remove particles. NAS-B and ASP-A were semi-prepared on a SHIMADZU LC-20A Prominence HPLC system using Venusil MP C18(2) (5 μm, 250 × 10 mm, Agela Technologies Inc.) at a flow rate of 3 mL $min^{-1}$. The MS and NMR data are summarized in **Supplementary Table 2-4** and the key HMBC and ROESY correlations in their structures are illustrated in **Supplementary Fig. 1-3**. The structures of NAS-B, ASP-A and NAS-E were deduced based on the NMR signals by following the strategy described previously[3].

### 3.5. Site-directed mutagenesis of $Nas_{F5053}$ and NascB

Rolling-circle PCR amplification was used to obtain all the site-directed mutants. Primers used for mutagenesis are all listed in **Supplementary Table 7**. Each PCR reaction (final volume 20 μL) containing 1 μL plasmid template, 1 μL primer pair (10 mM), 1 μL DMSO, 10 μL Primer Star DNA polymerase and 7 μL $ddH_2O$ were initiated at 98 °C for 30 s to denature the template DNA, followed by 30 amplification cycles. Each amplification cycle consisted of 98 °C for 10 s, 55 °C for 30 s and 72 °C for 3.30 min. The PCR cycles were finished with an extension step at 72 °C for 10 min. After analyzing by agarose gel electrophoresis, 5 μL of each PCR product was transformed respectively into *E. coli* DH5α. The transformed cells were spread on a Luria-Bertani

(LB) plate containing kanamycin and incubated at 37 °C overnight. Two colonies from each plate were grown and the plasmid DNA was isolated and verified by DNA sequencing.

Overlap-PCR was used to introduce mutations on multiple residues simultaneously. Taking fragment-7 of NascB as an example, two pairs of primers, nascB-F/nascB-7-R and nascB-7-F/nascB-R, were used to prepare two NDA fragments, respectively, based on the pWHU2485 DNA template. After purification by gel extraction kit, these two fragments were used as a template to amplify the whole nascB gene, by using nascB-F and nascB-R as primers. This PCR product was purified with the DNA gel extraction kit, and then cloned into the pET28a vector by the Gibson assembly kit. The plasmids were then isolated and sequenced to verify the desired mutations.

**3.6. Construction and screening of $Nas_{F5053}$ mutant libraries**

The 2-step PCR method developed by Reetz et al.[5] was applied to construct the $Nas_{F5053}$ library. With the pET21a-$Nas_{F5053}$ (wild-type) plasmid as the template, primers F5053-SM-NNK-F and F5053-SM-NNK-R were used to amplify the megaprimers. After all the megaprimers were confirmed by DNA agarose electrophoresis, they were used directly to amplify the whole plasmid. Templates were removed by *Dpn* I digestion at 37 °C for 7 h, which was confirmed by electrophoresis. PCR amplicons (2 µL) were directly transformed into 100 µL electrocompetent *E. coli* GB05-dir-T7, which contained the plasmid pWHU2487 expressing Fd and FdR. After adding 900 µL LB media, the cells were recovered at 37 °C for 1 h and then spread onto agar plates containing kanamycin (50 µg $mL^{-1}$), ampicillin (100 µg $mL^{-1}$) and apramycin (50 µg $mL^{-1}$).

After incubating for 14 h at 37 °C, 400 individual colonies were picked from the plate and inoculated into 500 µL of LB in a 2 mL 96-well plate. This plate was grown for 12-16 h at 37 °C and 220 rpm. 100 µL portions of each culture were transferred to a new 0.5 mL 96-well plate containing 100 µL of sterile glycerol (40 %, v/v) for stock. The rest of the bacteria were supplemented with 100 µmol IPTG, 400 µmol ALA and 200 µmol $(NH_4)_2Fe(SO_4)_2.6H_2O$ and continued to be expressed at 18 °C and 220 rpm for 20 h. The cells were harvested by centrifugation at 3,000 rpm at 4 °C and

resuspended in 400 μL M9 media. Then, 1 μL of $cW_L$-$P_L$ (100 mM) was added to the M9 media. After 48 h incubation at 18 °C, the reaction mixture was extracted with 1 mL ethyl acetate three times. The organic phase was transferred and dried by vacuum at a low temperature. Metabolites were subsequently redissolved by methanol and filtrated by a 0.45 μm membrane to remove particles. The activities of each mutants were analyzed by UPLC-MS, using the same condition mentioned above.

### 3.7. Synthesis of NAS-E

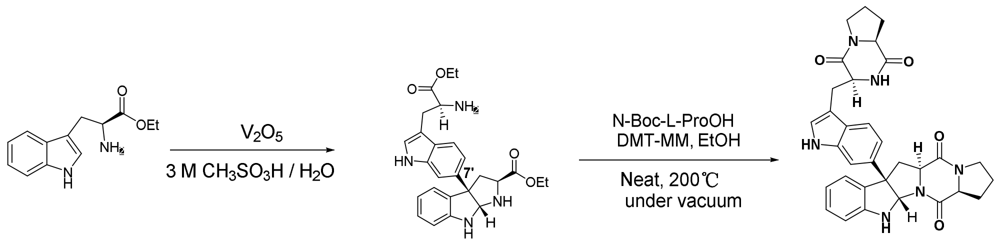


I

NAS-E (TM-3)

According to the method developed by Hayato Ishikawa[6], $V_2O_5$ (50.2 mg, 0.28 mmol) was added to an aqueous $CH_3SO_3H$ solution (10 mL, 3 M), and the suspended mixture was stirred at room temperature in an open flask until the solution became clear yellow. L−tryptophan ethyl ester (115.9 mg, 0.50 mmol) was added to the solution of $V_2O_5$ at 0 °C. The reaction mixture was stirred for 8 h at 0 °C in an open flask. The reaction mixture was slowly quenched with excess amount of aqueous 28% $NH_4OH$ solution at 0 °C. Then, the reaction was extracted three times with ethyl acetate. The combined organic layer was dried over $MgSO_4$, and concentrated under reduced pressure. Flash chromatography ($SiO_2$, 5% MeOH/ $CHCl_3$ to 10 % MeOH/ $CHCl_3$ and 20% MeOH/ EtOAc) provided the desired intermediate I: $^1$H NMR (400 MHz, $CDCl_3$) $\delta$ 8.02 (s, 1H), 7.46 (d, $J$ = 9.0 Hz, 1H), 7.19 (s, 1H), 7.08 (d, $J$ = 9.0 Hz, 1H), 7.04 (t, $J$ = 7.8 Hz, 1H), 6.98 (d, $J$ = 7.5 Hz, 1H), 6.95 (s, 1H), 6.67 (t, $J$ = 7.5 Hz, 1H), 6.56 (d, $J$ = 7.5 Hz, 1H), 5.18 (s, 1H), 4.20 (m, 4H), 3.78 (m, 2H), 3.18 (dd, $J$ = 4.8, 14.8 Hz, 1H), 2.98 (m, 1H), 2.80 (m, 1H), 2.59 (t, $J$ = 11.8 Hz, 1H), 1.25 (m, 6H); $^{13}$C NMR (101 MHz, $CDCl_3$) $\delta$ 175.1, 173.4, 149.6, 138.2, 136.0, 133.2, 128.0, 125.8, 124.9, 122.9, 119.2, 119.1, 118.0, 111.0, 109.4, 108.6, 85.9, 63.6, 61.3, 61.1, 60.6, 55.1, 46.0, 30.3, 14.2, 14.2.

To a solution of intermediate I (50 mg, 0.11 mmol) and N-(t-butoxycarbonyl)-L-proline (Boc-L-Pro, 47 mg, 0.22 mmol) in EtOH (5 mL), 4-(4, 6-dimethoxy-1,3,5-triazin-2-yl)-4-methylmorpholinium chloride (DMT-MM, 60 mg, 0.22 mmol) was added at 0 °C in an open flask (50 mL round-bottom flask). The resulting mixture was stirred for 4 h at 0 °C, before removing the solvent under reduced pressure. The crude material was directly heated at 200 °C under vacuum (0.1 mbar) for 15 min, before quenching with excess amount of aqueous 28% $NH_4OH$ solution. The aqueous layer was extracted three times with 5% MeOH/ $CHCl_3$. The combined organic layer was washed with brine, dried over $MgSO_4$, and concentrated under reduced pressure. Flash chromatography ($SiO_2$, 2 % MeOH/ saturated $NH_3$/ $CHCl_3$) provided NAS-E (TM-3) (10.0 mg) as white amorphous powder (**Supplementary Fig. 10**): $^1$H NMR (400 MHz, DMSO-*d6*) $\delta$ 10.79 (s, 1H), 7.65 (s, 1H), 7.50 (d, *J* = 8.4 Hz, 1H), 7.23 (s, 1H), 7.15 (d, *J* = 8.4 Hz, 1H), 7.14 (d, *J* = 2.7 Hz, 1H), 7.07 (dd, *J* = 1.5, 8.4 Hz, 1H), 6.99 (m, 1H), 6.66 (d, *J* = 3.0 Hz, 1H), 6.64 (d, *J* = 2.7 Hz, 1H), 6.61 (m, 1H), 5.56 (s, 1H), 4.31 (d, *J* =7.9 Hz, 1H), 4.26 (m, 1H), 4.17 (m, 1H), 4.05 (m, 1H), 3.34 (m, 2H), 3.25 (m, 2H), 3.20 (m, 1H), 3.04 (m, 1H), 3.00 (m, 1H), 2.65 (m, 1H), 2.13 (m, 1H), 1.96 (m, 2H), 1.80 (m, 2H), 1.60 (m, 2H), 1.38 (m, 1H); $^{13}$C NMR (101 MHz, DMSO-*d6*) $\delta$ 169.2, 166.2, 165.6, 165.4, 149.4, 136.9,136.0, 132.5, 128.3, 126.1, 124.9, 124.0, 119.0, 118.2, 116.4, 109.6, 109.2, 108.1, 81.5, 60.2, 59.8, 58.9, 58.5, 55.2, 44.7, 27.7, 27.4, 25.8, 22.6, 21.9.

### 3.8. Cloning, expression, crystallization and crystal structure determination of $Nas_{F5053}$ and its mutants

The first 5 amino acids (MTTTA) of $Nas_{F5053}$ were confirmed to have no effect on enzyme activity and were thus removed as presumably flexible, for potential benefit in crystallization. With the first 5 amino acids removed, the $Nas_{F5053}$ gene was amplified with the primer pair F5053-Xray-For/F5053-Xray-Rev and cloned into pSrtA9 through ligation-independent cloning[7]. Using New England Biolabs Q5 site-directed mutagenesis kit, the $Nas_{F5053}$-Q65I-A86G mutant was prepared through two successive rounds of single mutagenesis (the first primer pair: F5053-Q65I-For/

F5053-Q65I-Rev; the second primer pair: F5053-A86G-For/ F5053-A86G-Rev-). The $Nas_{F5053}$-S284A-V288A mutant was constructed with the primer pair $Nas_{F5053}$-S284A-V288A-For/$Nas_{F5053}$-S284A-V288A-Rev. The resulting constructs were confirmed by standard Sanger sequencing at the Australian Genome Research Facility (AGRF). $Nas_{F5053}$ was expressed as an N-terminal fusion with SrtA, i.e. His6-SrtA-$Nas_{F5053}$. The expression and purification were performed as previously reported[6] and further polished by running through a Superdex S200 gel filtration column with 20 mM HEPES, 100 mM NaCl, pH 7.5. As evaluated by SDS-PAGE, the elution fractions containing pure $Nas_{F5053}$ protein were pooled and concentrated to 7 mg $mL^{-1}$ for protein crystallization, using centrifugal filter units (Amicon MWCO 30 kDa, Millipore). The concentration of 7 mg $mL^{-1}$ was determined by absorbance at $A_{280}$ and a theoretical extinction coefficient. 1 mM DTT was added to the final $Nas_{F5053}$ protein solution. The yield of $Nas_{F5053}$ was approximately 1-2 mg $L^{-1}$ LB medium.Two extra amino acids, i.e. Gly and Ser, were left at the N-terminus of $Nas_{F5053}$.

Initial crystals were obtained in 0.2 M $CaCl_2$, 20% (w/v) polyethylene glycol (PEG) 3350at 20 °C, using the hanging drop vapor diffusion technique with the addition of 5% glycerol to the protein stock. The initial crystals were subsequently crushed for seeding by using the Seed Bead Kit (Hampton Research). The best crystals were obtained using the micro-seeding technique in 0.2 M $CaCl_2$, 22 % (w/v) PEG 3350, pH 7.5 at 4 °C. Substrate-bound protein crystals were obtained by soaking the substrate-free crystals in the mother liquor containing 2.5 mM $cW_L$-$P_L$ (diluting from 50 mM stock solution in DMSO) for 24 h.

Crystals were mounted onto CryoLoops (Hampton Research) and soaked in a cryoprotection solution containing 0.2 M $CaCl_2$, 22 % (w/v) PEG 3350, and 20% (v/v) glycerol prior to flash-cooling in liquid nigtrogen. For the substrate-bound protein crystals, the cryoprotection solution also contained 2.5 mM $cW_L$-$P_L$. X-ray diffraction data were collected at the Australian Synchrotron MX beamlines. The collected data were indexed and integrated using *XDS*[8] and scaled and merged using *Aimless*[9]. A partial initial model of the *ligand-free* structure was obtained by the molecular replacement technique with *Phaser* in *Phenix*[10] using the crystal structure of CYP121

from *Mycobacterium tuberculosis* (PDB ID **5WP2**) as the search model[11]. The initial model was improved by using the *Morph Model* tool in *Phenix*[12] and manually modified in *COOT*[13]. The substrate-bound structure was solved by the molecular replacement technique using the *ligand-free* structure as the search model. The structures were iteratively refined using *Phenix.Refine*[14] and manually modified in *COOT*.

### 3.9. Molecular dynamics simulations

***System preparation.*** Molecular dynamics simulations were performed based on the X-ray structures we determined in this study, including the wild-type $Nas_{F5053}$/$cW_L$-$P_L$ (PDB ID 6VXV), $Nas_{F5053}$-Q65I-A86G/$cW_L$-$P_L$ (PDB ID 6VZA), and $Nas_{F5053}$-S84A-V288A/$cW_L$-$P_L$ (PDB ID 6VZB). Parameters for substrates and the heme group for the molecular dynamics simulations were generated within the antechamber module of AMBER 18, using the general AMBER force-field, with partial charges set to fit the electrostatic potential generated at the B3LYP/6-31G(*d*) level by the restrained electrostatic potential model. The charges were calculated according to the Merz–Singh–Kollman scheme, using Gaussian 09. Amino acid protonation states were predicted using the H++ server (http://biophysics.cs.vt.edu/H++).

***Molecular dynamics simulation details***. The wild-type enzyme (PDB ID 6VXV) and variants were solvated in a pre-equilibrated truncated cuboid box with a 10 Å buffer of TIP4PEW water molecules, using the AMBER 18 leap module[15], resulting in the addition of approximately 9,000 solvent molecules. The system was neutralized by the addition of explicit counterions ($Na^+$ and $Cl^-$); in particular, we added 6 $Na^+$ counter ions. MD simulations were performed with the sander.MPI of Amber19 program using the Amber ff14SB force field for protein and GAFF2 for substrate[16]. The partial charges of the heme and the substrates were obtained at the B3LYP level of theory and LANL2DZ basis set for Fe and 6-31G for C, H, O, N, S atoms by Gaussian 09[17]. A two-stage geometry optimization approach was performed. The first stage minimizes the positions of solvent molecules and ions, imposing positional restraints on solute by a harmonic potential with a force constant of 500 kcal $mol^{-1}$ $Å^{-2}$, and the second stage is an unrestrained minimization of all of the atoms in the simulation cell. The systems are

gently heated using six 50 ps steps, incrementing the temperature 50 K each step (0–303 K, 30°C) under constant volume and periodic boundary conditions. Water molecules were treated with the SHAKE algorithm, such that the angle between the hydrogen atoms is kept fixed. Long-range electrostatic effects were modelled using the particle mesh Ewald method. An 8 Å cutoff was applied to Lennard-Jones and electrostatic interactions. Harmonic restraints of 10 kcal $mol^{-1}$ were applied to the solute and the Langevin equilibration scheme was used to control and equalize the temperature. The time-step was kept at 1 fs during the heating stages, allowing potential in homogeneities to self-adjust. Each system was then equilibrated without restrains for 2 ns, with a 2 fs time-step at a constant pressure of 1 atm and temperature of 300 K. After the systems were equilibrated in the NPT ensemble, 100 ns molecular dynamics simulations were performed under the NVT ensemble and periodic boundary conditions. The RMSDs of all the simulations are show in **Supplementary Fig. 14**.

### 3.10. UV-Vis titration and determination of the binding constants of $cW_L$-$P_L$ to $Nas_{F505}$, $Nas_{F5053}$-Q65I-A86G or $Nas_{F5053}$-S284A-V288A.

We used a double-beam UV-2600 (SHIMADZU) spectrophotometer and 1-cm pathlength quartz cells to measure UV-Vis absorbance of 2 μM $Nas_{F5053}$ or its mutants. Spectral titration with $cW_L$-$P_L$ was performed at 15 °C with reference to the reported procedures[18,19]. Difference spectra were generated by substracting the spectrum obtained without $cW_L$-$P_L$ from that recorded for each $cW_L$-$P_L$ concentration. Thereafter, the absorbance variations were calculated from the difference spectra ($\Delta A_{387}$-$\Delta A_{417}$) and were plotted against the relevant $cW_L$-$P_L$ concentrations. The binding constants (Ks) were extracted from fitting the plotted data points to the following equation:

$$\Delta A = (\Delta A_{\max} \cdot [S])/(K_s + [S])$$

where ΔA is the absorbane difference $\Delta A_{387}$-$\Delta A_{417}$, $\Delta A_{max}$ is the maximum reachable value of ΔA at saturating substrate concentrations, [S] is the substrate concentration and Ks is the apparent binding constant. Ks were reported as means of four independent experiments.

## 4. Supplementary References


1. Yu, H. & Li, S.-M. Two cytochrome P450 enzymes from Streptomyces sp. NRRL S-1868 catalyze distinct dimerization of tryptophan-containing cyclodipeptides. *Org. Lett.* **21**, 7094-7098 (2019).
2. Fan, X.-Y., Ma, H., Guo, J., Zhu, Z.-Q., Guo, S.-Q. & Zhao, G.-P. Development of mycobacterial inducible expression system and application for immunological diagnostics on tuberculosis. *Chin. J. Microbiol. Immunol.* ***29***, 1104-1109 (2009).
3. Tian, W., Sun, C., Zheng, M., Harmer, J. R., Yu, M., Zhang, Y., Peng, H., Zhu, D., Deng, Z., Chen, S.-L., Mobli, M., Jia, X. & Qu, X. Efficient biosynthesis of heterodimeric C-3-aryl pyrroloindoline alkaloids. *Nat. Commun.* ***9***, 4428 (2018).
4. Sambrook, J., Russell, D.W. Molecular cloning: a laboratory manual, 3rd ed. Cold Spring Harbor Laboratory Press, New York, (2001).
5. Li, A., Acevedo-Rocha C. G. & Reetz M. T., Boosting the efficiency of site-saturation mutagenesis for a difficult-to-randomize gene by a two-step PCR strategy. *Appl. Microbiol. Biotechnol.* ***102***, 6095-6103 (2018).
6. Tadano, S., Sugimachi, Y., Sumimoto, M., Tsukamoto, S. & Ishikawa, H. Collective synthesis and biological evaluation of tryptophan-based dimeric diketopiperazine alkaloids. *Chem. Eur. J.* ***22***, 1277-1291 (2016).
7. Jia, X., Crawford, T., Zhang, A. H. & Mobli, M. A new vector coupling ligation-independent cloning with sortase a fusion for efficient cloning and one-step purification of tag-free recombinant proteins. *Protein Expr. Purif.* ***161***, 1-7 (2019).
8. Kabsch, W. XDS. *Acta Cryst.* ***D66***, 125-132 (2010).
9. Evans, P. R. & Murshudov, G. N. How good are my data and what is the resolution? *Acta Cryst.* ***D69***, 1204-1214 (2013).
10. Adams, P. D., Afonine, P. V., Bunkóczi, G., Chen, V. B., Davis, I. W., Echols, N., Headd, J. J., Hung, L.-W., Kapral, G. J., Grosse-Kunstleve, R. W., McCoy, A. J., Moriarty, N. W., Oeffner, R., Read, R. J., Richardson, D. C., Richardson, J. S., Terwilliger T. C. & Zwart P. H. PHENIX: a comprehensive Python-based system for macromolecular structure solution. *Acta Cryst.* ***D66***, 213-221 (2010).
11. Fielding, A. J., Dornevil, K., Ma, L., Davis, I. & Liu, A. Probing ligand exchange in the P450 enzyme CYP121 from Mycobacterium tuberculosis: dynamic equilibrium of the distal heme ligand as a function of pH and temperature. *J. Am. Chem. Soc.* ***139***, 17484-17499 (2013).
12. Terwilliger, T. C., Read, R. J., Adams, P. D., Brunger, A. T., Afonine P. V. & Hung. L.-W. Model morphing and sequence assignment after molecular replacement. *Acta Cryst.* ***D69***, 2244-2250 (2013).

13. Emsley, P., Lohkamp, B., Scott, W. G. & Cowtan, K. Features and development of Coot. *Acta Cryst.* ***D66***, 486-501 (2010).
14. Afonine, P. V., Grosse-Kunstleve, R. W., Echols, N., Headd, J. J., Moriarty, N. W., Mustyakimov, M., Terwilliger, T. C., Urzhumtsev, A., Zwart P. H. & Adams, P. D. Towards automated crystallographic structure refinement with phenix.refine. *Acta Cryst.* ***D68***, 352-367 (2012).
15. Salomon-Ferrer, R., Case, D. A. & Walker, R. C. An overview of the Amber biomolecular simulation package. *Wires Comput. Mol. Sci.* ***3***, 198-210 (2013).
16. Maier, J.A., Martinez, C., Kasavajhala, K., Wickstrom, L. & Hauser K.E., Simmerling, C. ff14SB: Improving the accuracy of protein side chain and backbone parameters from ff99SB. *J. Chem. Theory Comput.* ***11***, 3696-3713 (2015).
17. Li, P. & Merz, K. M. Metal ion modeling using classical mechanics. *Chem. Rev.* ***117***, 1564-1686 (2017).
18. Schenkman, J., Jansson, I. Spectral analyses of cytochromes P450. *Methods Mol. Biol.* ***320***,11–18 (2006).
19. Belin, P. et al. Identification and structural basis of the reaction catalyzed by CYP121, an essential cytochrome P450 in Mycobacterium uberculosis. *Proc. Natl. Acad. Sci. U. S. A.* ***106***, 7426-7431 (2009).
