# Supplementary figures and images for: Molecular basis of regio- and stereo-specificity in biosynthesis of bacterial heterodimeric diketopiperazines
Source: Nat Commun. 2020 Dec 7;11:6251. doi: 10.1038/s41467-020-20022-5 (PMC7721796; doi:10.1038/s41467-020-20022-5)

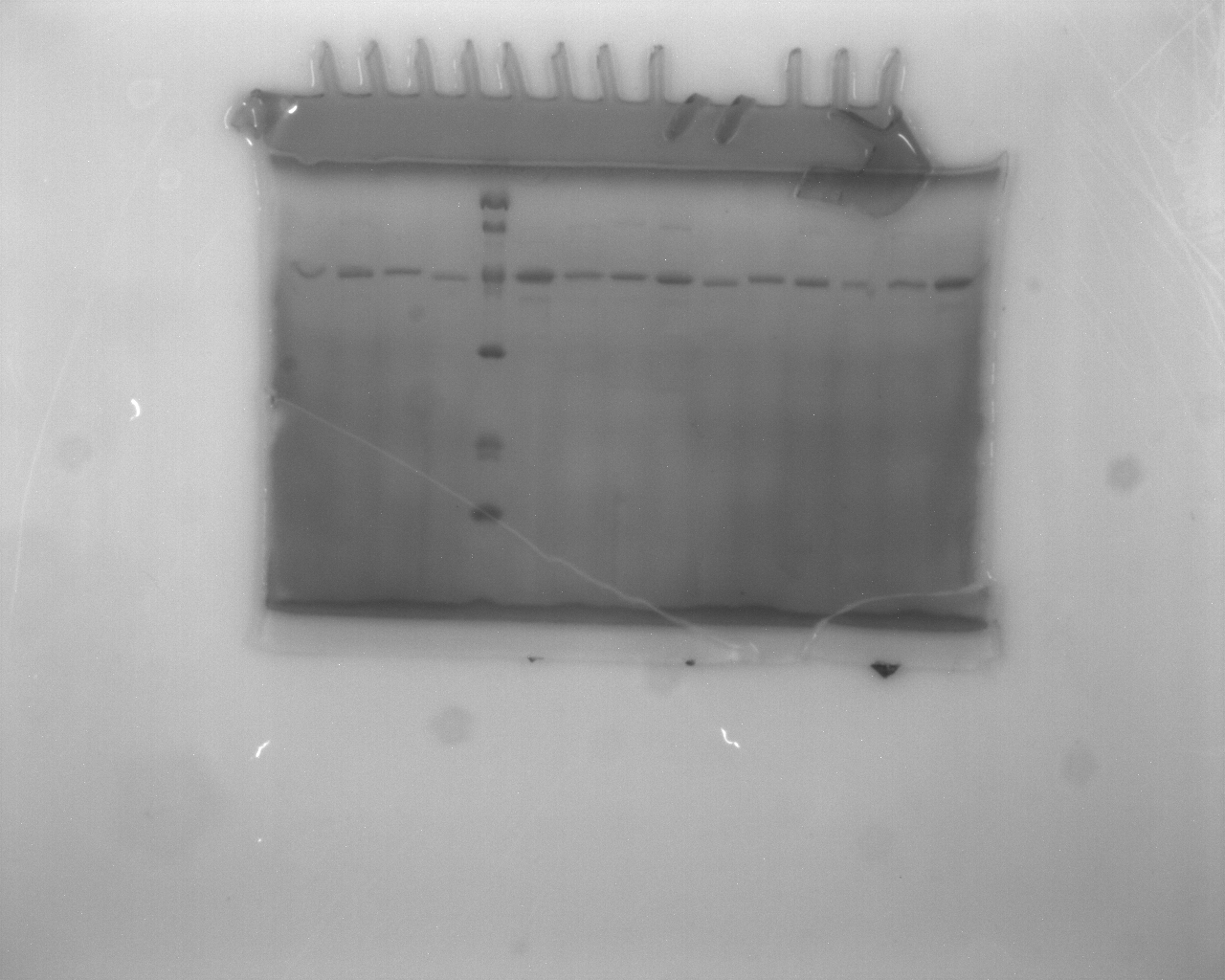

Supplement: Supplementary file 4 — Source Data [file 41467_2020_20022_MOESM4_ESM.zip › Source Data/Raw data of Supplementary Figure 15.jpg]
